# Iron-fueled ferroptosis: a new axis for immunomodulation to overcome cancer drug resistance—from immune microenvironment crosstalk to therapeutic translation

**DOI:** 10.3389/fimmu.2025.1726210

**Published:** 2026-01-13

**Authors:** Yimao Wu, Kaiyu Zhang, Naijun Jiang, Zichang Chen, Xiaojing Sun, Hongyu Zha, Mingjun Lin, Jingxin Li, Xiaocheng Pan, Jiadong Chen, Junbing He, Hongpeng Chen, Ruipei Chen

**Affiliations:** 1 Oncology, Jieyang People’s Hospital, Jieyang, China; 2 Second Clinical Medical College, Guangdong Medical University, Dongguan, China; 3 Second Clinical Medical College, Anhui Medical University, HeFei, China; 4 First Clinical Medical College, Anhui Medical University, Hefei, China; 5 Fourth Clinical Medical College, Anhui Medical University, Hefei, China; 6 Jieyang People’s Hospital, Jieyang, China; 7 Jieyang Medical Research Center, Jieyang People’s Hospital, Jieyang, China

**Keywords:** ferroptosis, cancer drug resistance, antitumor immunity, tumor microenvironment, immunotherapy, lipid peroxidation

## Abstract

Resistance to chemotherapy and targeted therapy in cancer is largely due to evasion of apoptosis, but ferroptosis—an iron-dependent form of regulated cell death driven by lipid peroxidation—offers a promising alternative, particularly in aggressive and therapy-resistant subtypes. The tumor immune microenvironment plays a central role in modulating ferroptosis susceptibility: CD8^+^ T cell-derived IFNγ downregulates system Xc^-^ and upregulates ACSL4, while other immune cells such as Tregs, MDSCs, and macrophages further fine-tune ferroptosis through cytokine and redox signaling. Importantly, ferroptosis induction promotes immunogenic cell death, enhancing T cell infiltration and synergizing with immune checkpoint blockade to achieve sustained antitumor immunity. This review delineates the molecular basis of ferroptosis sensitivity in resistant cancers, explores immune-ferroptosis crosstalk, evaluates combination strategies with immunotherapy, and discusses challenges such as toxicity and patient stratification to advance clinical translation.

## 1 Introduction

### 1.1 Clinical background

In cancer therapy, the development of drug resistance is a major factor limiting treatment efficacy. Both chemotherapeutic agents (such as cisplatin and paclitaxel) and targeted therapeutic drugs (such as EGFR inhibitors and BRAF inhibitors) can induce resistant phenotypes in cancer cells after prolonged use (1, 2). Drug resistance often stems from apoptosis evasion, but ferroptosis offers a tumor-specific alternative, particularly in malignancies with mesenchymal features (3). —— Cancer cells evade therapy-induced death signals by upregulating anti-apoptotic proteins (such as Bcl-2 and Mcl-1) or mutating apoptosis-related genes (such as p53), ultimately leading to treatment failure (4, 5).

### 1.2 The unique significance of ferroptosis

Ferroptosis was formally defined in 2012 as a form of regulated cell death that depends on iron ions and is triggered by the accumulation of lipid peroxides (6). Unlike traditional forms of cell death such as apoptosis, ferroptosis exhibits a unique sensitivity in drug-resistant cancer cells. On one hand, to adapt to therapeutic stress, resistant cancer cells undergo metabolic reprogramming, resulting in iron metabolism imbalance and the accumulation of lipid peroxidation substrates (7); On the other hand, their antioxidant systems, such as the glutathione–glutathione peroxidase 4 (GPX4) axis, often exhibit functional defects, making them incapable of eliminating excessive lipid peroxides. This selective vulnerability renders ferroptosis a potential breakthrough for overcoming cancer drug resistance (8).

For instance, in drug-resistant lung adenocarcinoma, ferroptosis sensitivity is markedly enhanced due to upregulated ACSL4 expression and metabolic reprogramming, which synergize with immune crosstalk—such as CD8+ T cell-derived IFN-γ—to overcome therapy resistance (9). This tumor-specific vulnerability provides a actionable target for combination strategies.

Notably, specific cancer subtypes—such as triple-negative breast cancer, lung adenocarcinoma, clear cell renal carcinoma, and mesenchymal-type tumors with high metastatic potential—exhibit heightened susceptibility to ferroptosis due to their inherent metabolic reprogramming, iron overload, and antioxidant system deficiencies. This selective vulnerability underscores the translational promise of ferroptosis-targeting strategies, offering a novel therapeutic avenue to overcome drug resistance and improve patient outcomes in these malignancies (10).

A deeper understanding of ferroptosis in drug-resistant cancers requires addressing several key questions: what is the molecular basis underlying the sensitivity of resistant cancer cells to ferroptosis (11)? How are the ferroptosis-related metabolic networks and signaling regulatory mechanisms organized (12)? How can these mechanisms be leveraged to develop effective and low-toxicity therapeutic strategies to reverse drug resistance (13)?

## 2 Ferroptosis susceptibility in drug-resistant cancers: core mechanistic frameworks

The clinical management of drug-resistant cancers has long been a central challenge in oncology. Notably, these phenotypic features—such as metabolic shifts in breast cancer models—interact dynamically with the immune microenvironment, enabling ferroptosis to serve as a lever for reversing resistance via pathways like IFN-γ signaling.Recent studies have revealed that most drug-resistant cancer cells—particularly subtypes with a mesenchymal phenotype and high metastatic potential—exhibit a uniquely heightened sensitivity to ferroptosis (14, 15). This sensitivity is not random; rather, it results from the interplay between the distinct molecular phenotypes acquired under prolonged drug selection pressure and adaptive metabolic alterations. These features are primarily manifested across three key dimensions: metabolic reprogramming, defects in antioxidant systems, and remodeling of the tumor microenvironment (16). A detailed analysis of these phenotypic features not only (14)facilitates a deeper understanding of the vulnerabilities of drug-resistant cancer cells but also provides a theoretical foundation for the development of ferroptosis-targeted therapies against resistant tumors (17).

### 2.1 Metabolic reprogramming features

Metabolic reprogramming enables tumor cells to adapt to proliferative demands and hostile microenvironments by remodeling key metabolic pathways. In drug-resistant cancer cells, this reprogramming is further skewed toward ferroptosis sensitivity. Enhanced glutaminolysis, remodeling of lipid composition, and disruption of iron homeostasis collectively provide ample “fuel” and “catalysts” for the initiation and execution of ferroptosis. This metabolic bias is not incidental; rather, it represents a trade-off acquired during the evolution of drug resistance in cancer cells (18).

#### 2.1.1 Enhanced glutaminolysis activity

Glutamine, as a conditionally essential amino acid for tumor cells, provides critical support for their survival and proliferation (19). Drug-resistant cancer cells enhance glutamine uptake by upregulating the glutamine transporter ASCT2 (SLC1A5).

Glutamine is subsequently converted to glutamate by glutaminase (GLS) and further deaminated to α-ketoglutarate (α-KG), which directly enters the mitochondrial matrix to participate in the tricarboxylic acid (TCA) cycle, providing energy for the cells (20). While the enhanced TCA cycle promotes ATP production via the electron transport chain (ETC), it also increases the electron leakage rates from mitochondrial complexes I and III (21, 22). These leaked electrons react with molecular oxygen to generate superoxide anions (O_2_•^-^), which are subsequently converted into hydrogen peroxide (H_2_O_2_) under the catalysis of superoxide dismutase (SOD) (23). In the presence of free iron, H_2_O_2_ undergoes the Fenton reaction to generate highly reactive hydroxyl radicals (•OH), providing a critical source o radicals to initiate lipid peroxidation chain reactions (24, 25). In addition, glutamine serves as a biosynthetic precursor, contributing to the synthesis of nucleotides, amino acids, and other essential biomolecules (26).

Studies have shown that in various drug-resistant cancer cells, including breast, lung, and colorectal cancers, glutaminolysis is upregulated, providing abundant reducing equivalents (NADH and FADH_2_) to the mitochondrial respiratory chain and thereby promoting the generation of reactive oxygen species (ROS) (27). The accumulation of ROS not only directly damages cellular macromolecules but also activates key metabolic enzymes through oxidative modifications, further driving metabolic reprogramming (28);Simultaneously, ROS serve as critical signaling molecules, activating a variety of oxidative stress–related pathways, upregulating the expression of ferroptosis-associated genes, and enhancing the potential for lipid peroxidation, thereby increasing the sensitivity of cancer cells to ferroptosis (29). For example, in drug-resistant breast cancer cells, inhibition of glutaminolysis significantly reduces intracellular ROS levels and diminishes the cytotoxic effects of ferroptosis inducers, such as RSL3 and Erastin (30). This “glutaminolysis–mitochondrial ROS–lipid synthesis” network collectively constitutes the metabolic basis for ferroptosis sensitivity in drug-resistant cancer cells.

#### 2.1.2 Lipidome remodeling

Dynamic remodeling of the lipidome represents another key metabolic feature underlying ferroptosis sensitivity in drug-resistant cancer cells. The functional axis composed of acyl-CoA synthetase long-chain family member 4 (ACSL4) and lysophosphatidylcholine acyltransferase 3 (LPCAT3) promotes the enrichment of polyunsaturated fatty acid phospholipids (PUFA-PLs) in cellular membranes, providing abundant substrates for lipid peroxidation (31). Drug-resistant cancer cells undergo lipidome remodeling to accumulate polyunsaturated fatty acid phospholipids (PUFA-PLs)—the critical “fuel” for ferroptosis—via the ACSL4-LPCAT3 axis. ACSL4, whose expression is 2.8-fold higher in triple-negative breast cancer (TNBC) resistant tumors (vs. sensitive ones), selectively converts PUFAs (e.g., AA, AdA) to PUFA-CoA; LPCAT3 then esterifies these intermediates into membrane-bound PE-AA/PE-AdA (PUFA-PLs). The bis-allylic hydrogen atoms in PUFA-PLs (bond dissociation energy ~88 kcal/mol) are highly susceptible to •OH-induced abstraction, triggering a self-amplifying lipid peroxidation chain reaction. This remodeling is particularly prominent in mesenchymal-like resistant cells, where ACSL4 upregulation is driven by YAP/TAZ signaling (activated via reduced cell-cell contact), further linking drug resistance to ferroptosis sensitivity. This reaction exhibits strict substrate specificity: ACSL4 has a 10–15-fold higher affinity for long-chain PUFAs containing more than 20 carbon atoms compared with saturated fatty acids, while it displays minimal catalytic activity toward short-chain fatty acids (32).

The resulting PUFA-CoA esters are subsequently recognized by LPCAT3, which catalyzes their esterification at the sn-2 position of lysophospholipids, such as lysophosphatidylethanolamine (LPE), ultimately generating PUFA-PLs that are incorporated into cellular and organelle membranes (33, 34).

The high abundance of PUFA-PLs enhances ferroptosis sensitivity because the bis-allylic hydrogen atoms within the PUFA chains (located between two carbon–carbon double bonds) have very low bond dissociation energies (~88 kcal/mol) and are readily abstracted by •OH or iron-catalyzed radicals, thereby initiating lipid peroxidation chain reactions (35). For example, the arachidonic acid (AA) chain in a PE-AA molecule can be attacked by radicals to form a phospholipid radical (PL•). PL• reacts with molecular oxygen to generate a phospholipid peroxyl radical (PLOO•), which then abstracts a hydrogen atom from a neighboring PUFA-PL to form a phospholipid hydroperoxide (PLOOH) while producing a new PL•, thereby perpetuating and amplifying the chain reaction (36).

High expression of ACSL4 directly increases the PUFA-PL content in cancer cell membranes by 2–3 fold, and this elevation is significantly positively correlated with ferroptosis sensitivity.

Clinical samples and model studies further validate the clinical significance of the ACSL4–LPCAT3 axis: in drug-resistant tumor tissues from patients with triple-negative breast cancer, ACSL4 expression levels were 2.8-fold higher than in sensitive tumors, and patients with high ACSL4 expression exhibited significantly enhanced responses to ferroptosis inducers combined with chemotherapy (37);In clear cell renal carcinoma cells, LPCAT3 expression is positively correlated with the levels of PE-AA and PE-AdA. Knockout of LPCAT3 markedly reduces the proportion of membrane PUFA-PLs and significantly decreases cellular sensitivity to GPX4 inhibitors (38).

#### 2.1.3 Iron homeostasis imbalance

Drug-resistant cancer cells, including cancer stem cells (CSCs), exhibit constitutive dysregulation of iron metabolism that prioritizes labile Fe²^+^ accumulation—an essential catalyst for ferroptosis. Unlike normal cells, which use the IRP1/IRP2-IRE system to restrict Fe²^+^ overload, resistant cells enhance Fe²^+^ availability via two non-redundant pathways (1): upregulation of transferrin receptor 1 (TfR1) (2–3-fold higher than sensitive cells) to increase iron uptake (2), activation of NCOA4-mediated ferritinophagy to degrade ferritin and release stored Fe³^+^ (subsequently reduced to Fe²^+^ by STEAP3). Recent studies confirm that this Fe²^+^ enrichment (40–60% higher LIP levels) creates a “pro-ferroptotic” state, as Fe²^+^ drives the Fenton reaction to generate hydroxyl radicals (•OH) and initiate lipid peroxidation (39). In contrast, drug-resistant cancer cells markedly elevate intracellular labile iron pool (LIP) levels by enhancing autophagy-mediated ferritin degradation (ferritinophagy) and transferrin receptor 1 (TfR1)-mediated iron uptake, providing abundant iron for the Fenton reaction and lipid peroxidation (40).

**Figure 1:**
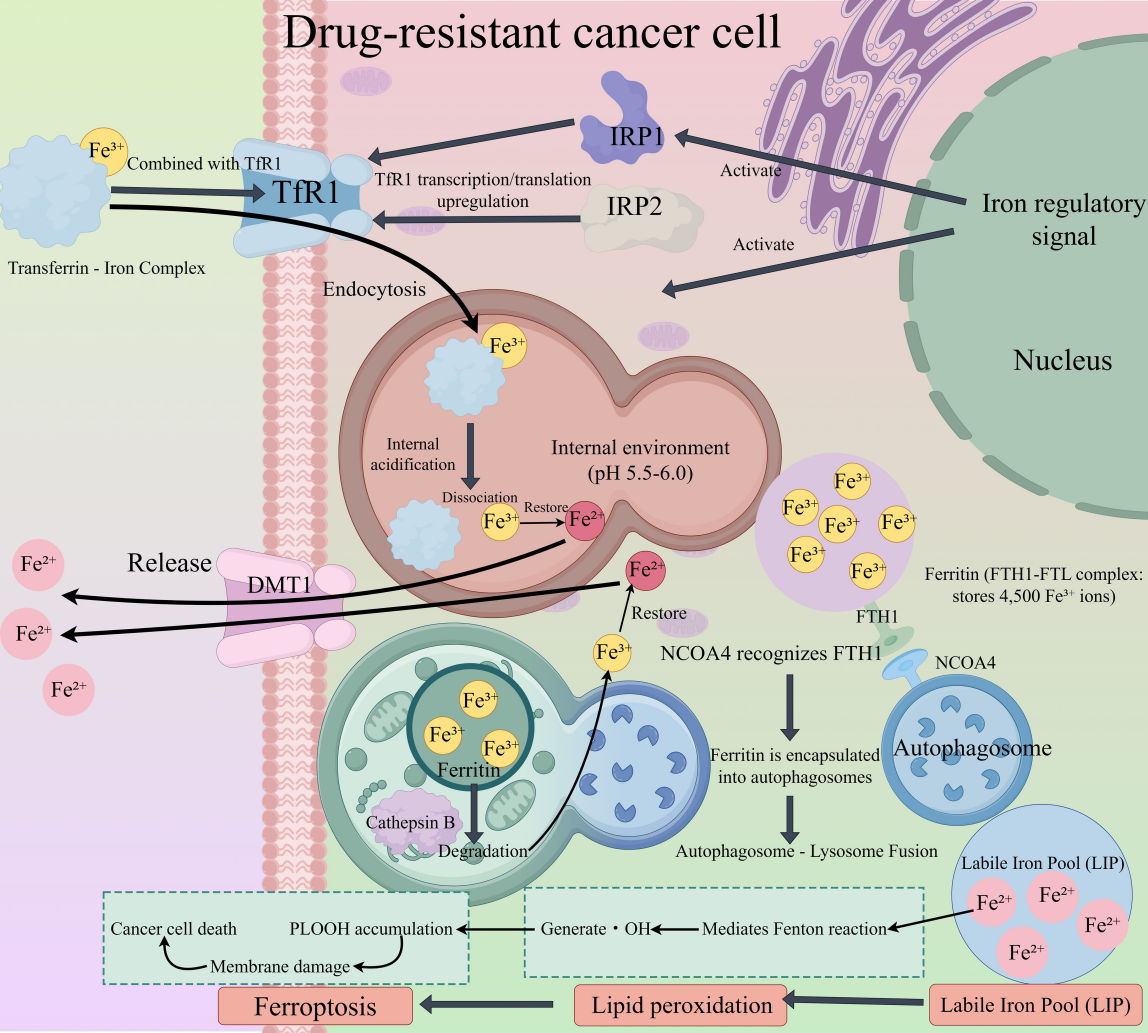
Schematic illustration of iron metabolism dysregulation in drug-resistant cancer cells leading to ferroptosis. Transferrin-bound Fe³^+^ binds to transferrin receptor 1 (TfR1) on the cell membrane; iron regulatory proteins (IRP1/IRP2) upregulate TfR1 transcription/translation, promoting endocytosis of the transferrin-iron complex. In the acidic endosomal environment (pH 5.5–6.0), Fe³^+^ dissociates, is reduced to Fe²^+^ by STEAP3, and is exported to the cytosol via DMT1. Meanwhile, nuclear receptor coactivator 4 (NCOA4) recognizes ferritin heavy chain 1 (FTH1) of the ferritin complex (FTH1-FTL, storing ~4,500 Fe³^+^ ions), mediating ferritin sequestration into autophagosomes. Autophagosome-lysosome fusion triggers ferritin degradation by cathepsin B, releasing additional Fe³^+^ (subsequently reduced to Fe²^+^). The accumulated Fe²^+^ expands the labile iron pool (LIP), mediating the Fenton reaction to generate hydroxyl radicals (•OH). These radicals induce lipid peroxidation, leading to phospholipid hydroperoxide (PLOOH) accumulation, membrane damage, and ultimately ferroptosis in drug-resistant cancer cells.

Ferritin is the primary intracellular iron storage protein, composed of 24 light and heavy chain subunits forming a hollow spherical structure capable of binding up to 4,500 Fe³^+^ ions (41). In drug-resistant cancer cells, the autophagy-related protein NCOA4 (nuclear receptor coactivator 4) is significantly upregulated. NCOA4 binds to the LC3-interacting region (LIR) of ferritin heavy chain (FTH1) and sequesters ferritin into autophagosomes. Upon fusion with lysosomes, ferritin is degraded in the acidic lysosomal environment (pH 4.5–5.0) by hydrolases such as cathepsin B, releasing Fe³^+^. This Fe³^+^ is subsequently reduced to Fe²^+^ by the lysosomal ferrireductase STEAP3 and exported into the cytosol via the divalent metal transporter DMT1 (SLC11A2). The resulting increase in the labile iron pool (LIP) markedly enhances free iron accumulation and ferroptosis sensitivity (42). The above mechanism can be seen in **Figure 1**.

Simultaneously, drug-resistant cancer cells enhance iron uptake by upregulating transferrin receptor 1 (TfR1, CD71). TfR1 is a key cell-surface receptor responsible for transferrin (Tf) binding and iron internalization, and its expression is regulated by IRP1/IRP2 (43). Under iron-deficient conditions, IRP1/IRP2 bind to the 3′ untranslated region (3′UTR) of TfR1 mRNA, stabilizing the transcript and promoting its translation (44). In drug-resistant cancer cells, the high metabolic activity increases iron demand, leading to persistent activation of IRP1/IRP2 and a 2–3-fold upregulation of TfR1 expression. Upon binding of transferrin-bound Fe³^+^ (Tf–Fe³^+^) in the bloodstream to TfR1, the complex is internalized via endocytosis. Following endosomal acidification, Fe³^+^ dissociates from transferrin, is reduced to Fe²^+^ by STEAP3, and subsequently released into the labile iron pool (LIP) via DMT1 (45). This multifaceted regulatory crosstalk renders iron homeostasis imbalance a “core amplifier” of ferroptosis sensitivity in drug-resistant cancer cells.

Notably, this Fe²^+^ accumulation is not accidental—it is a metabolic trade-off for drug resistance: resistant cells rely on increased iron to support rapid proliferation and DNA repair under therapeutic stress, but this dependence simultaneously amplifies their vulnerability to ferroptosis.

### 2.2 Defects in antioxidant systems

Drug-resistant cancer cells develop a “delicate and fragile balance” between enhanced antioxidant defenses and inherent vulnerabilities—this balance directly dictates ferroptosis susceptibility. While resistant cells upregulate the System Xc^-^-GSH-GPX4 axis to counteract oxidative stress (e.g., SLC7A11 overexpression in lung cancer), this defense is inherently fragile (1): System Xc^-^ activity is suppressed by CD8^+^ T cell-derived IFNγ (via JAK/STAT-mediated SLC7A11 downregulation), and (2) GPX4 function is dependent on GSH availability—any disruption to cystine uptake (e.g., erastin treatment) or GSH synthesis (e.g., glutaminolysis inhibition) rapidly inactivates GPX4. Importantly, CSCs and resistant subclones rely more heavily on this axis than sensitive cells, making their antioxidant defense “overloaded” and prone to collapse, thus conferring ferroptosis sensitivity (46). This defect is not a mere “loss of function” but represents an adaptive vulnerability acquired by cancer cells during the evolution of drug resistance to cope with metabolic stress.

#### 2.2.1 Impaired function or downregulation of system Xc^-^

The cystine–glutamate antiporter (System Xc^-^) is a critical “gateway” for maintaining intracellular glutathione (GSH) levels, and its impaired function or downregulation represents the most common form of antioxidant system defects in drug-resistant cancer cells. System Xc^-^ is a 1:1 heterodimer composed of SLC3A2 and SLC7A11 subunits, localized on the plasma membrane, and functions as an antiporter exchanging one molecule of extracellular cystine (Cys_2_) for one molecule of intracellular glutamate (Glu), thereby mediating cystine uptake. Once inside the cell, cystine is reduced to cysteine (Cys) by thioredoxin reductase 1 (TXNRD1) or glutathione reductase (GSR). Cysteine serves as the rate-limiting substrate for GSH synthesis, combining with glutamate and glycine under the catalysis of γ-glutamylcysteine synthetase (GCS) and glutathione synthetase (GS) to generate GSH (47, 48).

The direct consequence of transcriptional downregulation or functional inhibition of System Xc^-^ is insufficient GSH synthesis. As the most abundant intracellular non-protein thiol antioxidant, GSH not only serves as a crucial cofactor for GPX4 but also directly scavenges •OH and lipid radicals (49). Simultaneously, System Xc^-^ deficiency can trigger a vicious “oxidative stress–ER stress” cycle: GSH depletion disrupts the redox balance within the endoplasmic reticulum (ER), activating the unfolded protein response (UPR), which in turn further promotes ROS generation and exacerbates lipid peroxidation (50, 51). This cycle not only amplifies the defect in the antioxidant system but also provides a “dual driving force” for the initiation of ferroptosis. In summary, the impairment of System Xc^-^ function significantly enhances ferroptosis sensitivity in drug-resistant cancers, offering a promising therapeutic target for overcoming chemoresistance.Notably, certain drug-resistant cancer cells can counteract System Xc- inhibition by activating alternative cysteine acquisition pathways, such as the transsulfuration pathway mediated by cystathionine β-synthase (CBS) or direct cysteine uptake via ASCT transporters (52). Additionally, overexpression of glutamate-cysteine ligase (GCL) enhances *de novo* GSH synthesis, while upregulation of glutathione reductase (GR) promotes GSH regeneration from GSSG, collectively conferring resistance to ferroptosis inducers targeting the System Xc–GSH axis (53).

#### 2.2.2 Inhibition of GPX4 activity

Glutathione peroxidase 4 (GPX4), as the only intracellular enzyme capable of directly reducing lipid hydroperoxides (PLOOH), serves as the “last line of defense” against ferroptosis. Its inhibited activity is a key factor contributing to the increased ferroptosis sensitivity of drug-resistant cancer cells (54). GPX4 belongs to the selenoprotein family, with a selenocysteine (Sec) residue at its active site. Using GSH as an electron donor, GPX4 catalyzes the reduction of PLOOH to non-toxic phospholipid alcohols (PLOH), concurrently oxidizing GSH to glutathione disulfide (GSSG) (55, 56).

In normal cells, GPX4 expression and activity are maintained at relatively stable levels to cope with basal oxidative stress. In drug-resistant cancer cells, however, GPX4 activity is inhibited primarily through three mechanisms: downregulation of protein expression, modification of the active site, and deficiency of the cofactor GSH. Expression downregulation often arises from transcriptional or translational dysregulation. For example, under hypoxic conditions, HIF-2α indirectly suppresses GPX4 transcription by regulating two key intermediate molecules. In drug-resistant clear cell renal carcinoma cells, high HIF-2α expression is negatively correlated with GPX4 levels, promoting the formation of clear cell morphology and increasing ferroptosis sensitivity (38). Active site modifications are primarily mediated by specific inhibitors. For instance, the ferroptosis inducer RSL3 covalently binds to the Sec residue at GPX4’s active site, irreversibly inhibiting its activity (57, 58);FIN56 simultaneously promotes the ubiquitin-mediated degradation of GPX4 and binds to activate squalene synthase (SQS), leading to coenzyme Q10 (CoQ10) depletion and inhibition of the mevalonate pathway, thereby doubly impairing GPX4 function (59). Cofactor GSH deficiency is a direct consequence of System Xc^-^ impairment. As previously described, insufficient GSH deprives GPX4 of its electron donor, preventing effective reduction of PLOOH and ultimately leading to the accumulation of lipid peroxidation products (55). These findings not only validate the central role of GPX4 in ferroptosis sensitivity of drug-resistant cancers but also provide potential biomarkers for precision therapy. Thus, targeting GPX4 inhibition represents a viable strategy to induce ferroptosis and reverse drug resistance in various cancer types, with ongoing clinical trials exploring its therapeutic potential.

Conversely, some resistant cancers develop compensatory mechanisms to evade GPX4 inhibition, including GPX4 protein overexpression through NRF2-mediated transcriptional activation or Keap1 mutations, GPX4 Sec43Ser mutation that abolishes RSL3 binding, and enhanced selenium utilization that increases GPX4 synthesis and stability (60). Furthermore, alternative antioxidant systems such as the FSP1-CoQ10 and GCH1-BH4 pathways can compensate for GPX4 loss, significantly reducing ferroptosis sensitivity and contributing to therapeutic resistance (61).

### 2.3 Adaptive alterations in the tumor microenvironment

The tumor microenvironment (TME) is a complex ecosystem composed of cancer cells, stromal cells, immune cells, and the extracellular matrix (ECM). Its dynamic remodeling not only influences cancer cell proliferation and drug resistance but also modulates cellular metabolism and antioxidant systems, further amplifying ferroptosis sensitivity. Drug-resistant cancer cells adapt to TME features such as hypoxia, nutrient deprivation, and reduced cell–cell contact, forming a unique “microenvironment–cell phenotype” synergistic network. Key details of these phenotypic features and their underlying mechanisms are summarized in **Table 1**.

**Table 1:** Ferroptosis-sensitive phenotypic features of drug-resistant cancers.

Phenotypic category	Specific characteristics	Mechanistic basis	Representative examples	References
Metabolic Reprogramming	Enhanced glutaminolysis	ASCT2/GLS upregulation → glutamate/α-KG accumulation → mitochondrial ROS → Fenton reaction	TNBC, lung cancer (↑ROS by 2–3 folds)	(62, 63)
	Lipidome remodeling (high PUFA-PLs)	ACSL4/LPCAT3 activation → AA/AdA incorporation into membranes → lipid peroxidation substrate enrichment	TNBC (ACSL4 ↑2.8-fold vs. sensitive tumors)	(64, 65)
	Iron homeostasis imbalance	NCOA4-mediated ferritinophagy + TfR1 upregulation → labile iron pool (LIP) expansion	HCC, colorectal cancer (LIP ↑40%)	(66, 67)
Antioxidant System Defects	System Xc^-^ dysfunction (SLC7A11 downregulation)	Reduced cystine uptake → GSH depletion → GPX4 inactivation → lipid peroxide accumulation	NSCLC, ovarian cancer (GSH ↓50%)	(68, 69)
	GPX4 inhibition/downregulation	HIF-2α-mediated transcription suppression; RSL3-induced covalent modification of Sec residue	Clear cell RCC (GPX4 ↓60% under hypoxia)	(70, 71)
TME Adaptive Alterations	Hypoxic microenvironment	HIF-1α/HIF-2α → FASN/HILPDA upregulation → LD/PUFA-PL accumulation; NCOA4-mediated ferritinophagy	Solid tumors (lipid substrates ↑30% under hypoxia)	(72, 73)
	Reduced cell-cell contact (E-cadherin loss)	YAP/TAZ nuclear translocation → SLC7A11/GPX4 upregulation; FAK-PI3K-AKT → PUFA synthesis	Gastric cancer, breast cancer (EMT ↑70%)	(74, 75)

#### 2.3.1 Hypoxic microenvironment

A hypoxic microenvironment is a common feature of solid tumors, resulting from both increased oxygen consumption due to rapid tumor cell proliferation and impaired oxygen delivery caused by abnormal tumor vasculature (76, 77). Under hypoxic conditions, cancer cells activate hypoxia-inducible factor (HIF)–related pathways to initiate a series of adaptive responses, among which lipid metabolic reprogramming represents a key regulatory mechanism.

HIF-1α is a key transcription factor in the hypoxic response and can directly or indirectly regulate the expression of multiple genes involved in lipid metabolism, including fatty acid transport proteins (FATP), fatty acid–binding proteins (FABP), acetyl-CoA carboxylase (ACC), and fatty acid synthase (FASN). Upregulation of these genes enhances cancer cell uptake, synthesis, and esterification of fatty acids, thereby increasing intracellular triglyceride (TG) and phospholipid stores (78, 79). Peng and colleagues found that hypoxic trophoblast cells upregulate lnc-HZ06, which promotes SUMOylation of HIF-1α. This modification enables HIF-1α to bind the promoter region of nuclear receptor coactivator 4 (NCOA4), thereby upregulating NCOA4 transcription and protein expression. NCOA4 mediates ferritinophagy, increasing the labile iron pool and ultimately inducing ferroptosis in trophoblast cells, leading to miscarriage (80). Furthermore, HIF-1α enhances the transcription of the key glutamate transporter SLC1A1 and promotes cystine uptake, thereby contributing to ferroptosis resistance (81). This “increased lipid substrates–elevated iron catalysts” synergy renders the hypoxic microenvironment an important amplifier of ferroptosis sensitivity in drug-resistant cancer cells.

HIF-2α–mediated lipid droplet (LD) storage is a major mechanism underlying PUFA-PL accumulation under hypoxia. HIF-2α directly binds to the hypoxia response element (HRE) in the promoter region of the hypoxia-inducible lipid droplet–associated protein (HILPDA) gene, activating HILPDA transcription and promoting LD formation and maturation. As a key regulator of LDs, HILPDA inhibits adipose triglyceride lipase (ATGL) activity, reducing neutral lipid breakdown. Concurrently, it upregulates fatty acid synthase (FASN) and diacylglycerol acyltransferase (DGAT) expression, or enhances the function of lipid transport proteins such as FABP4, facilitating fatty acid accumulation in LDs and accelerating their formation (82). This storage is not a mere “lipid accumulation” but serves to reserve substrates for ferroptosis. Moreover, ROS generated under hypoxic conditions can further activate ferroptosis-related signaling pathways, synergistically enhancing cancer cell sensitivity to ferroptosis (83).

#### 2.3.2 Reduced cell–cell contact

Cell–cell contact is critical for maintaining tissue homeostasis. Intercellular communication mediated by adhesion molecules such as E-cadherin and β-catenin regulates cell proliferation, differentiation, and death (84). During tumorigenesis and progression, the patterns of contact between cancer cells often change, which can modulate ferroptosis-related molecule expression by affecting intracellular signaling pathways.

Studies have shown that when stable adhesions are formed between cells via E-cadherin, E-cadherin can interact with the core kinases of the Hippo pathway, indirectly promoting LATS1/2 phosphorylation and activation. This, in turn, phosphorylates YAP/TAZ, retaining them in the cytoplasm and rendering them inactive. In contrast, loss of E-cadherin and reduced cell–cell contact decrease LATS1/2 activity, leading to YAP/TAZ dephosphorylation and nuclear translocation. In the nucleus, YAP/TAZ bind TEAD family transcription factors to regulate the promoter regions of ferroptosis-suppressing genes, such as SLC7A11 and GPX4, thereby promoting their transcriptional expression (85); Simultaneously, β-catenin, which is bound to the intracellular tail of E-cadherin, is released from the plasma membrane and translocates into the nucleus, where it activates downstream target genes (86).

When cell–cell contact is reduced, epithelial cells may compensatorily enhance adhesion to the ECM, activating integrins and subsequently focal adhesion kinase (FAK). FAK further phosphorylates and activates the PI3K–AKT pathway, inhibiting apoptosis. However, sustained overactivation can upregulate enzymes involved in lipid synthesis, increasing the accumulation of polyunsaturated fatty acids (PUFAs) and providing molecular targets for ferroptosis (87). Similarly, Nrf2, the master transcription factor regulating antioxidant genes, is normally sequestered in the cytoplasm by Keap1. When reduced cell–cell contact enhances oxidative stress, excessive AKT activation may phosphorylate Keap1, promoting its degradation. However, sustained stress can lead to Nrf2 nuclear translocation fatigue, ultimately suppressing GPX4 expression, directly resulting in lipid peroxide accumulation and triggering ferroptosis (84).

#### 2.3.3 The role of other immune cells in regulating ferroptosis

Beyond CD8+ T cells, multiple immune cell populations within the tumor microenvironment (TME) critically influence ferroptosis sensitivity. Regulatory T cells (Tregs) can suppress ferroptosis by secreting anti-inflammatory cytokines such as IL-10, which inhibits lipid peroxidation and stabilizes antioxidant defenses in cancer cells, thereby promoting therapy resistance. This IL-10-mediated suppression is driven by activation of the STAT3 signaling pathway in cancer cells: IL-10 binds to its receptor (IL-10R) on cancer cell membranes, triggering STAT3 phosphorylation and nuclear translocation. Phosphorylated STAT3 then upregulates the transcription of antioxidant genes (e.g., GSTP1 and SLC7A11), reinforcing the glutathione-dependent antioxidant barrier and dampening ferroptosis. Notably, this Treg-IL-10-STAT3 axis not only protects cancer cells from ferroptosis but also sustains an immunosuppressive tumor microenvironment (TME)—a key immunomodulatory consequence that limits the efficacy of ferroptosis-based monotherapies. Myeloid-derived suppressor cells (MDSCs) contribute to ferroptosis resistance by producing arginase-1, which depletes arginine and reduces nitric oxide production, altering redox homeostasis and protecting tumors from iron-dependent death (88). Arginase-1-mediated arginine depletion not only impairs nitric oxide (NO)-dependent ferroptosis (via reduced NO-induced lipid peroxidation) but also suppresses the activation of CD8^+^ T cells—an essential component of antitumor immunity. This dual effect of MDSCs (ferroptosis resistance + T cell suppression) creates a feedforward loop that exacerbates immunological tolerance: reduced ferroptosis in cancer cells limits the release of immunogenic damage-associated molecular patterns (DAMPs), while arginine depletion directly blunts T cell-mediated cytotoxicity. Targeting this arginase-1-NO-ferroptosis axis thus represents a promising strategy to co-modulate immunometabolism and restore antitumor immunity. Natural killer (NK) cells can induce ferroptosis in target cells through the release of perforin and granzymes, which trigger mitochondrial dysfunction and enhance reactive oxygen species (ROS) generation, offering a complementary cytotoxic mechanism to T cell-mediated immunity (89). Mechanistically, perforin forms pores in cancer cell membranes to facilitate granzyme B entry, which then cleaves mitochondrial antiviral-signaling protein (MAVS)—a key adaptor of innate immune signaling. MAVS cleavage disrupts mitochondrial electron transport chain (ETC) integrity, increasing electron leakage and superoxide anion (O_2_•^-^) production. In the presence of labile iron (expanded in drug-resistant cancer cells), O_2_•^-^ is converted to hydroxyl radicals (•OH) via the Fenton reaction, accelerating lipid peroxidation. This NK cell-perforin-granzyme B-MAVS axis links innate immune cytotoxicity to ferroptosis, highlighting a novel immunomodulatory route to bypass apoptosis resistance in cancer. Macrophages, particularly the M2 phenotype, modulate ferroptosis by secreting transforming growth factor-beta (TGF-β), which promotes antioxidant responses and iron sequestration, TGF-β exerts its effects via activation of the Smad2/3 signaling pathway: upon binding to TGF-β receptors (TβRI/II) on cancer cells, Smad2/3 is phosphorylated and forms a complex with Smad4, which translocates to the nucleus to upregulate ferroportin (FPN1)—the primary iron export protein. Increased FPN1-mediated iron efflux reduces the labile iron pool (LIP), limiting Fenton reaction-driven lipid peroxidation. Concurrently, TGF-β/Smad signaling upregulates GPX4 expression, further reinforcing ferroptosis resistance. This M2-TGF-β-Smad-FPN1 axis underscores how immunosuppressive macrophages shape the TME to protect cancer cells from ferroptosis, emphasizing the need to target M2 polarization alongside ferroptosis induction for effective immunomodulation, thereby desensitizing cancer cells to ferroptosis inducers. This multifaceted regulation underscores the complexity of immune-ferroptosis crosstalk and highlights potential targets for combination therapies (90).

Collectively, these immune cell-ferroptosis interactions are not isolated regulatory events but integral to immunomodulation in drug-resistant cancers: CD8^+^ T cells and NK cells leverage ferroptosis to enhance antitumor immunity, while Tregs, MDSCs, and M2 macrophages exploit ferroptosis resistance to suppress immune responses. Dissecting these cross-talk mechanisms—such as IFNγ-JAK/STAT, IL-10-STAT3, and TGF-β-Smad pathways—provides a molecular basis to design combinatorial strategies (e.g., ferroptosis inducers + immune checkpoint inhibitors) that align ferroptosis activation with immunomodulation, ultimately overcoming therapy resistance.

Furthermore, the interplay between CD8+ T cells and other immune components, such as Tregs and macrophages, creates a dynamic network that can either amplify or suppress ferroptosis, providing new avenues for therapeutic intervention.

## 3 Core mechanisms driving ferroptosis in drug-resistant cancers

Unlike apoptosis, necrosis, and autophagy, ferroptosis exhibits distinct morphological, biochemical, and genetic characteristics (91). At the molecular level, multiple core regulatory systems exist: the classical glutathione peroxidase 4 (GPX4)–mediated system suppresses ferroptosis by clearing lipid peroxides, whereas the ferroptosis suppressor protein 1 (FSP1)–mediated system operates through the generation of radical-trapping antioxidants (92). Furthermore, the SMURF2–GSTP1 axis and the “sex hormone–MBOAT1/2–phospholipid remodeling” axis also contribute to ferroptosis regulation through distinct mechanisms, collectively forming the molecular regulatory network of ferroptosis in drug-resistant cancer cells (92, 93).

While separately detail iron metabolism and antioxidant defenses for organizational clarity, these systems are intrinsically interconnected in regulating ferroptosis sensitivity (94). Iron-driven lipid peroxidation and antioxidant responses function as complementary partners in a finely tuned regulatory network: iron metabolism provides the catalytic components (Fe2+) and lipid substrates (PUFA-PLs) necessary for peroxidation initiation, while antioxidant systems determine the threshold at which this peroxidation becomes lethal (95). Specifically, the labile iron pool (LIP) generated through ferritinophagy and TfR1-mediated uptake directly fuels the Fenton reaction, producing hydroxyl radicals that initiate lipid peroxidation; concurrently, the System Xc–GSH-GPX4 axis and FSP1-CoQ10 system work to contain this peroxidation within survivable limits (96). In drug-resistant cancers, the simultaneous upregulation of iron acquisition pathways and impairment of antioxidant defenses creates a perfect storm that dramatically lowers the threshold for ferroptosis induction, making these cells uniquely vulnerable to ferroptosis-based therapies.”

### 3.1 System Xc^-^–GSH–GPX4 axis: the classical ferroptosis defense system

Classical ferroptosis inducers target the System Xc^-^–GSH–GPX4 axis, which plays a central role in regulating the antioxidant system and lipid peroxidation (93), This pathway consists of three key molecules acting in series, collectively forming a robust antioxidant barrier (97). Inhibition of the System Xc^-^/GSH/GPX4 axis can effectively promote ferroptosis in cancer cells (46).

#### 3.1.1 System Xc^-^: The key “gateway” for cystine uptake

System Xc^-^ is a chloride-dependent, sodium-independent antiporter localized on the plasma membrane, composed of a light chain subunit SLC7A11 (xCT) and a heavy chain subunit SLC3A2 (CD98) in a 1:1 stoichiometric ratio (98). Among them, SLC7A11 serves as the functional transport subunit, specifically binding and importing extracellular cystine (Cys_2_) while exporting intracellular glutamate (Glu), achieving a 1:1 substrate exchange (99). As a key precursor for GSH synthesis, the efficiency of cystine uptake directly determines cellular antioxidant capacity and resistance to ferroptosis (100, 101).

In drug-resistant cancers, SLC7A11 expression is closely linked to ferroptosis sensitivity. For instance, in resistant models of lung and breast cancer, SLC7A11 is frequently overexpressed, enhancing cystine uptake to promote GSH synthesis and thereby increasing cancer cell resistance to ferroptosis (102, 103). Conversely, inhibition of SLC7A11 transport function can significantly impair System Xc^-^ activity and promote ferroptosis (104). Currently, three mechanisms regulating SLC7A11 function have been identified. The first involves small-molecule inhibitors, such as erastin and sulfasalazine, which directly suppress transport activity by binding to specific sites on SLC7A11 (105, 106); The second mechanism involves competitive inhibitors that occupy the substrate-binding site of SLC7A11, thereby interfering with cystine uptake (106); The third mechanism operates at the genetic level: transcription factors such as ATF3 and p53 can directly repress SLC7A11 transcription, while microRNAs, such as miR-26b, inhibit SLC7A11 translation by targeting its mRNA, ultimately downregulating System Xc^-^ function (107, 108).

#### 3.1.2 Glutathione: The major intracellular water-soluble antioxidant

GSH is the primary water-soluble antioxidant within cells and an essential cofactor for glutathione peroxidase 4 (GPX4) function. Cystine imported via SLC7A11 is reduced intracellularly to cysteine, which subsequently participates in GSH synthesis (46). GSH plays a crucial role in maintaining intracellular homeostasis, particularly in response to oxidative stress and toxic insults (46). One of its primary functions is to catalyze the reduction of lipid peroxides to non-toxic alcohols via glutathione peroxidases (GPXs), thereby protecting cells from oxidative damage (109). Furthermore, the balance between the reduced (GSH) and oxidized (GSSG) forms of glutathione is regulated by various intracellular enzymes, such as glutathione reductase, whose activity directly affects GSH availability (109). Thus, intracellular GSH levels directly determine the capacity to scavenge lipid peroxides and serve as a critical “metabolic barrier” enabling drug-resistant cancer cells to resist ferroptosis.

#### 3.1.3 Glutathione peroxidase 4 (GPX4)

In drug-resistant cancer cells, GPX4 expression and activity are generally elevated. Its expression is not only influenced by upstream GSH levels but also regulated by signaling pathways such as mTORC1 (46, 110). For example, mTORC1 can induce GPX4 protein synthesis via the Rag-mTORC1-4EBP axis (111). Additionally, KLF11 can suppress GPX4 transcription by binding to its promoter region, thereby reducing cellular resistance to ferroptosis (112). PX4 activity is also indirectly regulated by GSH availability: when System Xc^-^ function is impaired, leading to insufficient GSH synthesis, GPX4 lacks the necessary electron donor to effectively eliminate PLOOH, resulting in the accumulation of lipid peroxides.

Upregulation of the System Xc^-^–GSH–GPX4 axis represents a central strategy by which drug-resistant cancer cells resist ferroptosis. Inhibition of any component of this pathway—such as blocking SLC7A11 with erastin or directly inhibiting GPX4 with RSL3—can effectively induce ferroptosis in resistant cancer cells (110, 113).

In the GPX4 catalytic cycle, GPX4–SeH is oxidized by PLOOH to GPX4–SeOH, and GSH reduces SeOH to further activate GPX4, releasing GSSG and preventing GPX4 inactivation. GSSG is reduced to GSH by the cofactor NADPH. As PLOOH is reduced to PLOH by GPX4–SeH, ferroptosis is inhibited.

### 3.2 FSP1–CoQ10–NAD(P)H pathway: the second line of cellular defense against ferroptosis

The FSP1–CoQ10–NAD(P)H pathway is the second major ferroptosis-inhibiting system independent of GPX4. It operates in parallel with the classic System Xc^-^–GSH–GPX4 axis, together forming a “dual barrier” against lipid peroxidation in cells (114). This pathway consists of three core components: ferroptosis suppressor protein 1 (FSP1, formerly AIFM2), coenzyme Q10 (CoQ10), and NAD(P)H (115). Its uniqueness lies in being GSH-independent, providing an alternative ferroptosis defense mechanism for drug-resistant cancer cells (116).

#### 3.2.1 FSP1 regulation of ferroptosis

FSP1 is a flavin-containing oxidoreductase. The N-terminal myristoylation site in its structure allows FSP1 to localize to subcellular structures such as the plasma membrane and Golgi apparatus, exerting antioxidant effects in close proximity (117). Functionally, FSP1 uses NAD(P)H as an electron donor to reduce ubiquinone (CoQ10) to ubiquinol (CoQ10H_2_) (118). Ubiquinol, as a lipid-soluble radical-trapping antioxidant, can directly bind lipid peroxyl radicals (PLOO◼), blocking the lipid peroxidation chain reaction (116).

Recent studies show that FSP1 regulation also involves non-classical substrates and metabolic pathways. For example, FSP1 can efficiently reduce vitamin K (phylloquinone, menaquinone) to its hydroquinone form (VKH_2_), which also possesses potent radical-trapping capacity, inhibiting (phospho)lipid peroxidation (119). Additionally, FSP1 expression is negatively correlated with lipid synthesis enzymes ACSL4 and LPCAT3; by indirectly downregulating their activity, FSP1 reduces the accumulation of polyunsaturated fatty acid phospholipids (PUFA-PLs) in the cell membrane, lowering ferroptosis sensitivity (120). SP1 also functionally interacts with the dihydroorotate dehydrogenase (DHODH) pathway, another mitochondrial CoQ reductase, which can partially resist ferroptosis (120).

#### 3.2.2 FSP1 regulatory mechanisms: from metabolism to post-translational modifications

FSP1 function is regulated at multiple levels including cellular metabolism, transcriptional control, and post-translational modifications. Metabolically, as an NAD(P)H-dependent oxidoreductase, FSP1 activity is closely linked to cellular energy metabolism. Cancer cells with high glycolytic activity (e.g., tumors with pronounced Warburg effect) have elevated NADH levels, providing sufficient electron donors for FSP1 and enhancing its anti-ferroptotic capacity. Conversely, inhibition of glycolysis or mitochondrial oxidative phosphorylation can limit NAD(P)H supply, weakening FSP1 function (121).

At the transcriptional level, NRF2 and BACH1 are key regulators. NRF2, as the central transcription factor for antioxidant responses, can directly bind antioxidant response elements (AREs) in the FSP1 promoter to upregulate its expression. BACH1 competitively binds ARE sites, inhibiting NRF2, downregulating FSP1 expression, thereby promoting ferroptosis (122).

Post-translational modifications of FSP1 are also critical. TRIM21, an E3 ubiquitin ligase, mediates K63-linked ubiquitination at FSP1 residues K322 and K366, promoting FSP1 membrane translocation and enhancing anti-ferroptotic activity (117). FSP1 stability is further regulated by m6A RNA methylation: YTHDC1 recognizes m6A sites in the 3’UTR of FSP1 mRNA, recruiting CSTF3 to mediate selective polyadenylation, generating unstable short 3’UTR FSP1 mRNA. When YTHDC1 is downregulated, HuR-stabilized long 3’UTR FSP1 mRNA is formed, resulting in increased FSP1 protein levels (123).

#### 3.2.3 Parallel operation of the FSP1–CoQ10–NAD(P)H pathway with other systems

Although the FSP1–CoQ10 system can independently exert anti-ferroptotic effects apart from GPX4, under physiological conditions, these two systems usually work synergistically, providing multi-layered antioxidant defense (124). Spatially, FSP1 primarily localizes to the plasma membrane, generating CoQ10H_2_ to protect membrane lipids from oxidative damage, while GPX4 is broadly distributed in mitochondria, ER, and other organelle membranes, clearing PLOOH in subcellular compartments (116). This division allows precise protection against lipid peroxidation in different regions, explaining why FSP1 expression levels are negatively correlated with ferroptosis sensitivity; cancer cells with high FSP1 expression often exhibit stronger resistance to GPX4 inhibitors (125).

Beyond GPX4, FSP1 intersects with multiple antioxidant systems. ALDH7A1 can generate membrane-bound NADH to supply electrons for FSP1 while consuming reactive aldehydes to reduce lipid peroxidation (121). Interestingly, ferroptosis stress activates AMP-activated protein kinase (AMPK), promoting ALDH7A1 membrane localization and further stabilizing FSP1 membrane association, forming a positive feedback loop (121). Another cooperative mechanism involves heme oxygenase-1 (HO-1): HO-1 degradation increases ferroptosis sensitivity, whereas HO-1 overexpression reduces ROS and lipid peroxidation accumulation, inhibiting ferroptosis (126). HO-1 may indirectly affect FSP1 function by regulating intracellular iron levels, as it is the key enzyme for heme degradation (126). Additionally, ferritin heavy chain 1 (FTH1) has functional connections with FSP1; miR-375-3p can simultaneously downregulate FTH1 and FSP1 expression, jointly promoting ferroptosis (127). The FSP1-mediated pathway provides an alternative mechanism to suppress ferroptosis, particularly in cancers with GPX4 resistance, highlighting its clinical relevance as a complementary target for combination therapies.

### 3.3 The SMURF2–GSTP1 axis: a third major ferroptosis regulatory system

The SMURF2–GSTP1 axis is a newly identified ferroptosis regulatory pathway, whose core mechanism involves the dynamic balance between ubiquitin–proteasome–mediated protein degradation and antioxidant activity, providing a novel perspective for understanding GPX4-independent ferroptosis regulation (93). Under basal conditions, glutathione S-transferase P1 (GSTP1) maintains cellular redox homeostasis through selenium-independent antioxidant activity. Upon ferroptosis-inducing signals, SMAD-specific E3 ubiquitin ligase 2 (SMURF2) mediates the ubiquitination and degradation of GSTP1, weakening the antioxidant defense and ultimately triggering ferroptosis (128).

#### 3.3.1 GSTP1: an important endogenous inhibitor of ferroptosis

GSTP1, a core member of the GST superfamily, exerts dual antioxidant functions (129, 130). First, GSTP1 possesses selenium-independent glutathione peroxidase activity, directly neutralizing lipid peroxides (93). Second, GSTP1 catalyzes the conjugation of toxic lipid peroxidation products such as 4-hydroxynonenal (4-HNE) with GSH, facilitating detoxification and excretion of these harmful compounds (128).

In pancreatic cancer, GSTP1 has been shown to protect cells from radiation-induced ferroptosis (131). Radiation upregulates ACSL4 expression to induce ferroptosis, while GSTP1 overexpression reduces ACSL4 levels and increases GSH content, thereby enhancing cancer cell resistance to irradiation (131). Similarly, in doxorubicin-treated breast cancer cells, GSTP1 promotes autophagy to counteract drug-induced ferroptosis (132). These findings indicate that GSTP1 serves as a key mediator of therapy resistance across multiple cancer types.

Notably, GSTP1 expression is regulated by multiple factors. The small antigen (s-HDAg) of hepatitis delta virus (HDV) specifically binds GSTP1 mRNA and suppresses its expression, leading to ROS accumulation and cell death (133). At the transcriptional level, NRF2 acts as the primary positive regulator of GSTP1 expression (134), whereas AP-1 may suppress its transcription (135). Clinically, these factors play important roles in inhibiting the antioxidant function of the SMURF2–GSTP1 axis and promoting ferroptosis in cancer cells.

#### 3.3.2 Molecular characteristics of SMURF2 and its role in protein homeostasis

SMURF2, a key member of the HECT-type E3 ubiquitin ligase family, exerts its central role in ferroptosis regulation by recognizing and degrading GSTP1 (136, 137). Upon ferroptosis-inducing stimuli (e.g., oxidative stress or GPX4 inhibition), SMURF2 becomes activated and binds GSTP1 via its HECT domain, mediating K48-linked ubiquitination. The ubiquitinated GSTP1 is then recognized and degraded by the proteasome, resulting in a sharp decline in antioxidant capacity and accumulation of lipid peroxides (93). This mechanism establishes the SMURF2–GSTP1 axis as the third major ferroptosis regulatory system, alongside GPX4 and FSP1.

Beyond GSTP1 regulation, SMURF2 can also ubiquitinate and degrade Dishevelled (DVL) proteins, thereby modulating the Wnt signaling pathway (136). This pathway is closely associated with cellular metabolism and oxidative stress, and its aberrant activation may enhance ferroptosis resistance by upregulating SLC7A11 expression. In addition, SMURF2 participates in regulating HIF-1α stability, influencing tumor hypoxia adaptation and metabolic reprogramming, thereby indirectly affecting ferroptosis sensitivity (137).

#### 3.3.3 Cooperation and competition between SMURF2 and other ubiquitination systems in ferroptosis regulation

The ubiquitin–proteasome system (UPS) consists of various E1, E2, and E3 enzymes, and different E3 ligases can act cooperatively or antagonistically in ferroptosis regulation by targeting the same or different substrates.

NEDD4L (NEDD4-like E3 ubiquitin ligase) suppresses ferroptosis by ubiquitinating and degrading lactotransferrin (LTF), thereby reducing intracellular iron accumulation (138). LTF, a key iron-transporting protein that binds and transfers Fe³^+^, upon degradation, limits extracellular iron uptake, lowers the substrate concentration for the Fenton reaction, and consequently decreases ROS generation and lipid peroxidation (138); In contrast, SMURF2 promotes lipid peroxide accumulation by degrading the antioxidant protein GSTP1. The two ligases thus target distinct ferroptosis control points — iron metabolism (NEDD4L) and antioxidant defense (SMURF2) — and may cooperatively amplify ferroptotic signaling under specific stress conditions (138, 139). This cooperation appears tumor-type specific: in iron overload–induced ferroptosis, NEDD4L-mediated inhibition of iron uptake predominates; whereas under oxidative stress–induced ferroptosis, the SMURF2–GSTP1 axis plays a more crucial role. Moreover, SMURF2 and NEDD4L expression may be co-regulated by upstream stress signaling, allowing simultaneous activation to intensify ferroptotic responses.

#### 3.3.4 Hippo-YAP pathway regulation of ACSL4 transcription

Beyond the canonical GPX4, FSP1, and GSTP1 pathways, emerging evidence implicates the Hippo-YAP signaling axis as a key regulator of ferroptosis resistance through transcriptional control of ACSL4. Yes-associated protein (YAP), the primary effector of the Hippo pathway, directly binds to TEAD transcription factors and activates ACSL4 promoter activity, enhancing ACSL4 mRNA and protein expression (93).This upregulation increases polyunsaturated fatty acid phospholipid (PUFA-PL) synthesis, thereby amplifying lipid peroxidation and ferroptosis sensitivity. Conversely, Hippo pathway activation via LATS1/2 phosphorylates and inactivates YAP, leading to nuclear exclusion and reduced ACSL4 transcription, which confers ferroptosis resistance (140). This mechanosensitive pathway integrates cellular stress signals with lipid metabolic reprogramming, providing a novel therapeutic target for modulating ferroptosis in drug-resistant cancers.

### 3.4 “Sex hormone–MBOAT1/2–phospholipid remodeling” axis: a novel pathway regulated by sex hormones

The “sex hormone–MBOAT1/2–phospholipid remodeling” axis represents a ferroptosis regulatory pathway independent of the above three antioxidant mechanisms. The surveillance mediated by GPX4, FSP1, and GSTP1 acts by reducing PL hydroperoxides and terminating PL oxidation chains at specific stages of PL peroxidation (46, 92). Unlike the above mechanisms, MBOAT1/2 reduce the amount of PL–PUFA (polyunsaturated fatty acids) by increasing PL–MUFA (monounsaturated fatty acids), thereby decreasing PL–PUFA levels upstream (92).

#### 3.4.1 Biological functions of MBOAT1 and MBOAT2

MBOAT1/2 are membrane-bound O-acyltransferases that inhibit ferroptosis by remodeling the composition of cellular membrane phospholipids. Specifically, MBOAT2 selectively transfers monounsaturated fatty acids (MUFA) to lysophosphatidylethanolamine (lyso-PE), thereby increasing cellular PE–MUFA and correspondingly reducing PE–PUFA (polyunsaturated fatty acids) (92, 141).

MBOAT1, also known as LPEAT1, transfers oleic acid (OA) to lysophosphatidylethanolamine (lyso-PE) and lysophosphatidylserine (lyso-PS). Similar to MBOAT2, MBOAT1 suppresses ferroptosis through PL remodeling and preferentially promotes PE–MUFA formation (92). In addition, MBOAT1 significantly reduces RSL3-induced lipid peroxidation, which may result from the reduction of PE–AA (phosphatidylethanolamine bearing an arachidonoyl moiety) (92).

#### 3.4.2 Regulatory mechanisms of sex hormones on MBOAT1/2

The expression of MBOAT2 is regulated by the androgen receptor (AR) and may serve as a potential therapeutic target in cancers such as prostate cancer (92, 141, 142). Androgens regulate MBOAT2 expression through the AR signaling pathway. As a ligand-activated transcription factor, AR directly binds to androgen response elements (AREs) in the MBOAT2 gene to enhance its transcription, and the use of the androgen antagonist Enzalutamide can inhibit this effect (92, 143).

Estrogens upregulate MBOAT1 expression and activity through multiple mechanisms. In the genomic pathway, estrogen receptors (ER) α and β act as transcription factors that directly bind to estrogen response elements (EREs) in the promoter region of the MBOAT1 gene to enhance its transcription (92, 144). In the non-genomic pathway, activation of the membrane-bound G protein–coupled estrogen receptor (GPER) promotes MBOAT1 expression via the ERK/MAPK signaling cascade (145, 146). Furthermore, estrogens can indirectly influence MBOAT1 activity by altering the cellular redox state, as the enzyme is sensitive to oxidative stress (92). Experimental evidence shows that MBOAT1 expression is significantly elevated in ER-positive breast cancer cells, inhibiting ferroptosis and promoting tumor growth; treatment with estrogen antagonists such as Fulvestrant can block this effect and restore cellular sensitivity to ferroptosis (92).

Beyond the four core regulatory axes detailed above, the translation of ferroptosis induction into clinical strategies for drug-resistant cancers requires explicit linking of pathways to targeted agents, applicable tumor types, and their practical advantages and limitations—an integration that is systematically summarized below.

#### 3.4.3 systematically integrates three key pathways with direct relevance to drug-resistant cancer cell lines

First, the System Xc^-^-GSH-GPX4 axis remains the most well-characterized target for overcoming drug resistance. Inhibitors of this axis act through two primary strategies: blocking cystine uptake via System Xc^-^ or directly inactivating GPX4. Erastin and sulfasalazine are classic System Xc^-^ inhibitors, effective in reversing cisplatin resistance in non-small cell lung cancer (NSCLC) and paclitaxel resistance in triple-negative breast cancer (TNBC) cell lines (147). These agents exhibit high specificity for cancer cells with downregulated System Xc^-^ (a common phenotype in resistant subtypes), minimizing off-target effects on normal cells. However, their clinical application is limited by poor water solubility and short half-lives, requiring frequent dosing to maintain therapeutic concentrations. Direct GPX4 inhibitors, such as RSL3 and ML210, show potent activity in drug-resistant hepatocellular carcinoma (HCC) and pancreatic ductal adenocarcinoma (PDAC) cells—particularly those with acquired resistance to tyrosine kinase inhibitors (TKIs). A key advantage of GPX4 inhibitors is their ability to bypass adaptive upregulation of System Xc^-^, a major resistance mechanism to erastin-like agents. Nevertheless, they often induce dose-dependent renal and hepatic toxicity due to GPX4 expression in normal tissues (148).

Second, the iron homeostasis regulatory pathway offers a tumor-selective route to induce ferroptosis in resistant cells. Agents targeting this pathway include deferoxamine (DFO, an iron chelator that disrupts iron storage) and dihydroartemisinin (DAT, which triggers lysosomal ferritin degradation). DAT has shown promise in reversing carboplatin resistance in ovarian cancer cell lines by expanding the labile iron pool (LIP) and enhancing Fenton reaction-mediated lipid peroxidation (149). Its advantage lies in synergistic effects with conventional chemotherapies, as iron overload amplifies chemotherapy-induced oxidative stress. However, DAT requires careful monitoring of systemic iron levels to avoid anemia, and its efficacy is reduced in cancer cells with upregulated ferroportin (an iron export protein). Another agent, lactotransferrin (LTF) inhibitors (e.g., NEDD4L agonists), reduce iron import in doxorubicin-resistant colorectal cancer (CRC) cells, but their use is restricted by variable LTF expression across tumor types.

Third, the ACSL4-LPCAT3 lipid remodeling pathway is critical for sensitizing mesenchymal-like drug-resistant cancer cells. Sorafenib, a multi-kinase inhibitor that indirectly upregulates ACSL4, effectively reverses TKI resistance in renal cell carcinoma (RCC) and HCC cell lines by increasing polyunsaturated fatty acid phospholipid (PUFA-PL) accumulation (150). A unique advantage of sorafenib is its dual role in inhibiting oncogenic signaling (e.g., VEGFR) and promoting ferroptosis, addressing both tumor growth and drug resistance. However, sorafenib often induces adaptive activation of the Nrf2 antioxidant pathway, which upregulates GPX4 and counteracts ferroptosis. Additionally, its efficacy is limited in cancer cells with low ACSL4 expression, such as luminal subtype breast cancer (151). Summary of Currently Used Ferroptosis-Inducing Agents Across Cancer Types can be seen in **Table 2**.

**Table 2:** Summary of currently used ferroptosis-inducing agents across cancer types.

Agent name	Mechanism of action	Applicable tumor types	References
Erastin	Inhibits System Xc^-^ (SLC7A11/SLC3A2 complex), blocking cystine uptake → GSH depletion → GPX4 inactivation	Non-small cell lung cancer (NSCLC, cisplatin-resistant), triple-negative breast cancer (TNBC, paclitaxel-resistant)	(152, 153)
RSL3	Covalently binds to the selenocysteine residue of GPX4 → irreversible inhibition of GPX4 activity	Hepatocellular carcinoma (HCC, TKI-resistant), pancreatic ductal adenocarcinoma (PDAC, drug-resistant)	(154–156)
Sulfasalazine	Competitively inhibits System Xc^-^, reducing cystine import → impaired GSH synthesis	Colorectal cancer (CRC, doxorubicin-resistant), NSCLC (immune checkpoint inhibitor-resistant)	(157, 158)
Dihydroartemisinin (DAT)	Triggers lysosomal ferritin degradation (non-autophagic pathway) → expands labile iron pool (LIP) → enhances Fenton reaction	Ovarian cancer (carboplatin-resistant), CRC (5-FU-resistant)	(159, 160)
Sorafenib	Indirectly upregulates ACSL4 → increases PUFA-PL accumulation; inhibits VEGFR signaling	Renal cell carcinoma (RCC, TKI-resistant), HCC (sorafenib-naive/resistant)	(54, 161)
ML210	Directly binds to GPX4’s active site → suppresses lipid peroxide clearance	Melanoma (BRAF inhibitor-resistant), TNBC (platinum-resistant)	(162, 163)

## 4 Therapeutic strategies targeting ferroptosis in drug-resistant cancer

Based on the mechanisms of ferroptosis, current research has revealed that ferroptosis is closely associated with various diseases at both organ and tissue levels, such as cardiomyopathy and intervertebral disc degeneration (164, 165). Researchers have identified multiple targeted pathways to inhibit or promote ferroptosis, offering new hope for treating various diseases (166, 167). In particular, in the treatment of drug-resistant cancers, ferroptosis-targeting strategies have shown great potential, including monotherapy, combination therapy, and optimization of delivery systems, aiming for more precise and sensitive cancer treatment (168).

### 4.1 Monotherapy strategies

#### 4.1.1 Inhibition of cellular antioxidant systems

Trace element selenium, as a core molecule in ferroptosis regulation, plays an irreplaceable role in maintaining cellular redox homeostasis (169). Selenium participates in the synthesis of glutathione peroxidase 4 (GPX4), a key negative regulator of lipid peroxidation, which efficiently removes intracellular lipid peroxides and thereby maintains membrane stability (54). Cystine, the dimeric form of cysteine, is specifically transported into cells through system Xc^-^ (the cystine/glutamate antiporter), subsequently enhancing GPX4 catalytic activity to effectively inhibit ferroptosis (170). Multiple studies have confirmed that when system Xc^-^ function is inhibited, intracellular lipid peroxidation levels increase exponentially, triggering ferroptosis (101).

For example, researchers such as L. Zhou et al. found that targeting inhibition of ZDHHC8 protein activity significantly reduces GPX4 palmitoylation levels in cancer cells, blocking intracellular antioxidant defenses and markedly enhancing tumor cell sensitivity to ferroptosis (171). Erastin and RSL3, as classical ferroptosis activators, act through different mechanisms: the former inhibits system Xc^-^, blocking cystine uptake and indirectly suppressing GPX4 by reducing glutathione synthesis (172);the latter directly and covalently inhibits GPX4, leading to lipid peroxide accumulation (173). However, both agents suffer from poor water solubility, low stability, and high off-target toxicity, severely limiting clinical application. Therefore, developing highly efficient targeted systems is of great significance, although clinical translation remains challenging (174).

#### 4.1.2 Regulation of intracellular iron metabolism

As an iron-dependent form of regulated cell death, ferroptosis is closely linked to intracellular iron homeostasis—regulating iron metabolism pathways and molecules can directly influence cellular ferroptosis sensitivity (6). Due to their rapid proliferation, cancer cells exhibit significantly higher iron uptake and utilization capacity than normal cells, making them more susceptible to ferroptosis-inducing signals and providing a theoretical basis for targeting iron metabolism in cancer therapy (175).

The level of iron uptake, transferrin receptor 1 (TfR1)-mediated iron import is the primary pathway, whereas iron export is mainly controlled by ferroportin. Enhancing iron import protein function or inhibiting iron export activity increases intracellular labile iron levels, thereby heightening ferroptosis sensitivity in cancer cells (176). In addition, transient receptor potential mucolipin 1 (TRPML1), a non-selective cation channel localized on lysosomal membranes, participates in maintaining iron balance through regulation of lysosomal exocytosis (177). In cancers with hyperactivated protein kinase B (AKT), AKT phosphorylates TRPML1 at Ser343, inhibiting its ubiquitination and degradation, thereby stabilizing TRPML1’s interaction with ADP-ribosylation factor–like GTPase 8B (ARL8B). This triggers lysosomal exocytosis and promotes iron efflux, ultimately conferring resistance to ferroptosis (178). This mechanism positions TRPML1 as a potential anticancer target: TRPML1-competitive peptides can block its interaction with ARL8B, inhibit lysosomal exocytosis, increase intracellular iron accumulation, directly promote ferroptosis, and enhance tumor cell sensitivity to ferroptosis inducers, radiotherapy, and immunotherapy, thereby offering a new strategy for cancer therapy targeting ferroptosis (179).

Beyond protein-level regulation, intracellular iron content is finely tuned at the transcriptional level via the iron-responsive element/iron regulatory protein (IRE–IRP) system (39). Under iron-deficient conditions, iron regulatory proteins (IRP1 and IRP2) bind to iron-responsive elements (IREs) within mRNAs, modulating iron homeostasis through two mechanisms: (i) binding to the 5′UTR of mRNAs encoding iron export proteins, thus repressing their translation and reducing iron efflux (180); and (ii) binding to the 3′UTR of TfR1 mRNA to stabilize the transcript and enhance TfR1 expression, promoting iron uptake (44). Targeted inhibition of the IRE–IRP system disrupts iron homeostasis in cancer cells and may overcome drug resistance by inducing ferroptosis. For instance, Chen et al. demonstrated that dihydroartemisinin (DAT) triggers lysosomal degradation of ferritin via a non-autophagic pathway, elevating the cellular labile iron pool. Concurrently, DAT enhances IRP binding to IRE-containing mRNAs, further modulating IRP/IRE-mediated iron homeostasis and promoting free iron accumulation—offering a novel transcriptional-level approach for single-agent ferroptosis induction (181).

#### 4.1.3 Regulation of lipid peroxidation

Phospholipid peroxidation is the defining biochemical event in the execution of ferroptosis, and the fatty acid composition of membrane phospholipids directly determines susceptibility to oxidative damage (6). It is well established that membranes enriched in polyunsaturated phospholipids (PUFA–PLs) are particularly prone to peroxidation (182). This vulnerability arises because the bis-allylic hydrogen atoms in PUFA structures possess relatively low bond energies, making them susceptible to abstraction by reactive oxygen species (ROS) or iron-catalyzed radicals. This initiates a lipid peroxidation chain reaction that ultimately compromises membrane integrity and induces cell death (183).

Within the molecular network governing PUFA–PL synthesis and ferroptosis sensitivity, ACSL4 catalyzes the conjugation of long-chain polyunsaturated fatty acids with coenzyme A (CoA) to form PUFA–CoA esters. Subsequently, LPCAT3 re-esterifies these PUFA–CoA intermediates into lysophospholipids, producing PUFA–PLs that are integrated into cellular membranes (33). This “ACSL4 activation–PUFA–CoA formation–LPCAT3-mediated esterification” axis dictates the proportion of PUFA–PLs within membranes: elevated ACSL4 expression or activity increases PUFA–PL abundance, thereby heightening ferroptosis sensitivity. Conversely, genetic knockout or pharmacological inhibition of ACSL4 replaces long-chain PUFA–PLs with monounsaturated phospholipids (MUFA–PLs), allowing cells to survive for months even under ferroptosis-inducing conditions such as GPX4 depletio (184).

Zhang et al. further identified protein kinase CβII (PKCβII) as a “sensor molecule” for lipid peroxidation. Upon the generation of initial lipid peroxides, PKCβII is activated and directly phosphorylates ACSL4 at Thr328, enhancing its catalytic activity toward PUFA–PL synthesis. This establishes a “lipid peroxidation–PKCβII–ACSL4” positive feedback loop that amplifies ferroptosis-associated lipid damage signals. In tumor cells with high PKCβII and ACSL4 expression, targeting this feedback axis efficiently triggers ferroptosis, providing a promising therapeutic strategy for drug-resistant cancers (185).

### 4.2 Combination therapy strategies

#### 4.2.1 Integration with conventional cancer treatments

The conventional cancer treatment system is anchored by surgery, radiotherapy, and chemotherapy, and also encompasses adjuvant modalities such as hormone therapy and targeted therapy. Its fundamental goal is to achieve tumor control by directly killing tumor cells or inhibiting their proliferation. In the field of radiotherapy, the antitumor effects of high-energy ionizing radiation are primarily mediated through two mechanisms: first, it directly damages the DNA structure of tumor cells, leading to genetic material impairment and blocking cell division (186); second, it ionizes intracellular water molecules, triggering a series of chemical reactions and generating large amounts of reactive oxygen species (ROS), which further exacerbate oxidative stress-induced cellular damage (187).

However, the existence of radioresistance severely limits the efficacy of radiotherapy. Particularly in the treatment of hepatocellular carcinoma (HCC), radioresistance is a key factor contributing to treatment failure. Therefore, the development of therapeutic strategies that can effectively reverse radioresistance and enhance the radiosensitivity of tumor cells has become an urgent need to improve the prognosis of HCC patients. Recently, Chen Qianping et al. identified suppressor of cytokine signaling 2 (SOCS2) from differentially expressed genes using RNA sequencing (RNA-seq) combined with bioinformatics analysis, and confirmed that it can serve as a potential prognostic predictor for HCC radiotherapy. Mechanistic studies demonstrated that SOCS2 directly binds to SLC7A11 (a critical subunit of System Xc^-^) via its E3 ubiquitin ligase activity, thereby promoting the K48-linked ubiquitination and degradation of SLC7A11. This process impairs the function of System Xc^-^; reduced System Xc^-^ activity then inhibits cystine uptake, which in turn decreases glutathione (GSH) synthesis. As a key cofactor for glutathione peroxidase 4 (GPX4), the depletion of GSH results in the loss of GPX4’s antioxidant activity, ultimately triggering the accumulation of lipid peroxidation and ferroptosis (188). This finding not only fills the research gap regarding the role of SOCS2 in ferroptosis in HCC but also provides novel biomarkers and therapeutic targets for precision radiotherapy in HCC, bearing significant value for basic research and potential for clinical translation.

#### 4.2.2 Integration with immunotherapy

Interferon-γ (IFNγ) produced by T cells, in conjunction with arachidonic acid (AA), can cooperatively induce immunogenic ferroptosis in tumor cells. This mechanism constitutes a key effector pathway of CD8^+^ T cell–mediated cytotoxicity, providing a molecular bridge between cancer immunotherapy and ferroptosis-targeted treatment (189). IFNγ​ enhances tumor ferroptosis sensitivity through two distinct mechanisms. In triple-negative breast cancer, for example, IFN-γ-mediated downregulation of SLC7A11 not only potentiates ferroptosis but also enhances CD8+ T cell infiltration, creating a positive feedback loop that is absent in SLC7A11-low colorectal cancers, highlighting the need for tumor-specific biomarker stratification (190).First, IFNγ directly stimulates the expression and activity of ACSL4, remodeling the lipid composition of cancer cell membranes by increasing polyunsaturated phospholipid (PUFA–PL) content, thereby rendering cells more susceptible to ferroptosis (189); Second, IFNγ activates the Janus kinase/signal transducer and activator of transcription (JAK/STAT) pathway, leading to transcriptional and translational downregulation of the two critical System Xc^-^ subunits, SLC3A2 and SLC7A11. This suppression impairs cystine transport, enhances lipid peroxidation, and drives ferroptotic progression (191).

Beyond CD8^+^ T cells and IFNγ, emerging evidence underscores the contributions of macrophages, dendritic cells, neutrophils, and myeloid-derived suppressor cells (MDSCs) to ferroptosis modulation. For instance, tumor-associated macrophages (TAMs) can polarize toward an M2-like phenotype upon encountering ferroptotic debris, secreting cytokines that either sensitize or resist ferroptosis in a context-dependent manner (192). Dendritic cells enhance antigen presentation and T-cell priming when exposed to ferroptosis-derived damage-associated molecular patterns (DAMPs), while neutrophils release reactive oxygen species (ROS) and proteases that amplify lipid peroxidation. MDSCs, by contrast, often suppress ferroptosis through upregulation of antioxidant pathways like GPX4, thereby fostering an immunosuppressive microenvironment (193). Integrating these insights can refine combination therapies to target multiple immune axes simultaneously.

**Figure 2:**
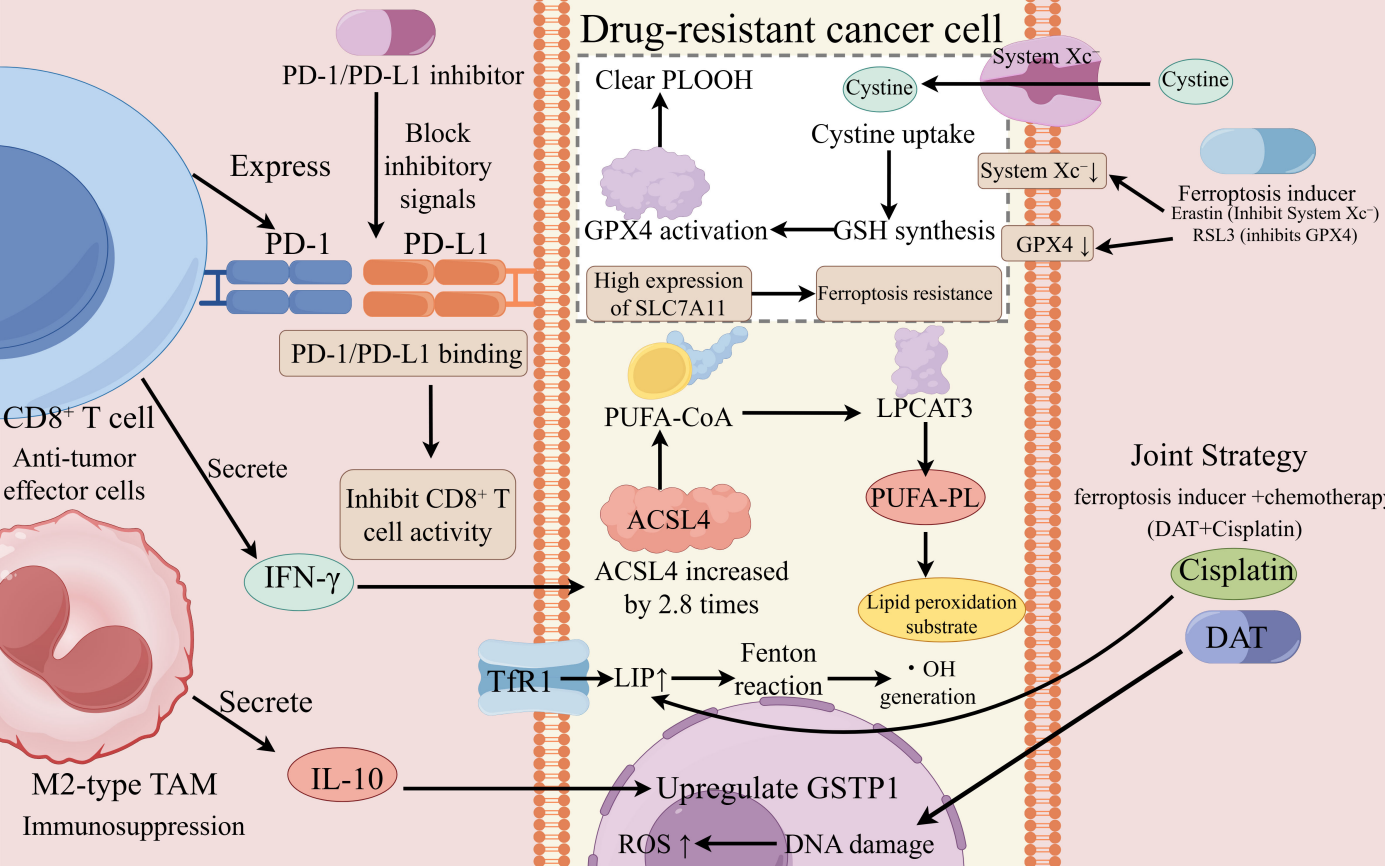
Synergistic mechanisms of PD-1/PD-L1 inhibitors combined with ferroptosis inducers (and chemotherapy) against drug-resistant cancer. Left: PD-L1 overexpression on drug-resistant cancer cells binds to PD-1 on CD8^+^ T effector cells, transmitting inhibitory signals to suppress T cell activity. PD-1/PD-L1 inhibitors block this interaction, restoring CD8^+^ T cell function—activated CD8^+^ T cells secrete IFNγ, which upregulates ACSL4 (by 2.8-fold) to promote PUFA-CoA synthesis. LPCAT3 esterifies PUFA-CoA into PUFA-PLs (lipid peroxidation substrates), enhancing ferroptosis sensitivity. Middle: Ferroptosis inducers target key antioxidant pathways—Erastin inhibits System Xc^-^ (blocking cystine uptake, reducing GSH synthesis), while RSL3 directly inhibits GPX4 (impairing PLOOH clearance). Drug-resistant cancer cells with high SLC7A11 expression exhibit ferroptosis resistance, which is reversed by the combined action of immunotherapy and ferroptosis inducers. Right: Immunosuppressive factors in the tumor microenvironment—M2-type tumor-associated macrophages (TAMs) secrete IL-10 to upregulate GSTP1 (enhancing antioxidant defense, suppressing ferroptosis). Bottom: Joint chemotherapy strategy (DAT + Cisplatin): DAT expands the labile iron pool (LIP) via TfR1 to promote Fenton reaction-mediated •OH generation; Cisplatin induces DNA damage and ROS accumulation, synergizing with ferroptosis to kill drug-resistant cancer cells.

Programmed death-ligand 1 (PD-L1), encoded by the CD274 gene and belonging to the B7 family of immune checkpoint proteins, is frequently overexpressed in the tumor microenvironment, facilitating immune evasion. By binding to its receptor programmed death-1 (PD-1) on T cells, PD-L1 transmits inhibitory signals that suppress T-cell activation and cytotoxicity (194). PD-1/PD-L1 inhibitors disrupt this pathway, restoring CD8^+^ T cell–mediated antitumor immunity and exhibiting remarkable therapeutic efficacy in immunogenic cancers. Importantly, PD-L1 downregulation can positively modulate T-cell function by stimulating CD8^+^ T cells to secrete IFNγ. The released IFNγ, in turn, enhances tumor ferroptosis sensitivity via the mechanisms described above, establishing a synergistic “immune activation–ferroptosis induction” feedback axis that underpins the integration of immunotherapy and ferroptosis-based strategies (195). The above mechanism can be seen in **Figure 2**.

ACSL4 upregulation not only enhances cellular susceptibility to ferroptosis but also promotes immunogenic cell death (ICD), which directly facilitates CD8^+^ T cell infiltration and activation. ACSL4-mediated membrane lipid peroxidation triggers the release of damage-associated molecular patterns (DAMPs), such as ATP, HMGB1, and calreticulin, which act as chemoattractants and activation signals for dendritic cells (196). This process enhances antigen presentation and primes CD8^+^ T cells, leading to increased tumor infiltration and IFNγ secretion. IFNγ further upregulates ACSL4 expression via the JAK/STAT pathway, creating a positive feedback loop that amplifies both ferroptosis and anti-tumor immunit (197).

This regulatory network establishes an integrated synergistic framework for overcoming therapeutic resistance. Specific combinations of ferroptosis inducers with conventional or immunotherapies, along with their synergistic mechanisms and efficacy, are detailed in **Table 3**. PD-L1 inhibitors restore T-cell functionality, enabling direct immune-mediated cytotoxicity while simultaneously promoting IFNγ secretion to suppress System Xc^-^ activity in tumor cells (198). Building upon this immune activation, combination therapy with ferroptosis inducers such as Erastin and RSL3 markedly strengthens ferroptosis induction. Specifically, Erastin blocks cystine import by inhibiting System Xc^-^, whereas RSL3 directly inactivates GPX4. Together, these agents synergize with IFNγ-driven regulation to achieve robust ferroptosis activation and immunogenic tumor cell death, offering a promising paradigm for overcoming drug resistance in cancer therapy (54, 199).

**Table 3:** Combination therapeutic strategies targeting ferroptosis.

Combination type	Combination regimen	Mechanistic synergy	Applicable drug-resistant cancer types	Efficacy (preclinical/clinical)	Advantages	Disadvantages	References
Ferroptosis Inducer + Radiotherapy	Erastin + Ionizing Radiation (IR)	IR upregulates SOCS2 → SLC7A11 degradation → GSH depletion; Erastin blocks System Xc^-^ → synergistic lipid peroxidation	HCC (radiation-resistant), NSCLC (cisplatin-radiation resistant)	HCC: Radiosensitivity ↑35%; SOCS2^+^ tumors: Pathological complete response (pCR) ↑28%	Enhances radiation-induced oxidative stress; targets radioresistant subclones	Erastin’s poor water solubility requires frequent dosing	(200, 201)
	RSL3 + Stereotactic Body Radiation Therapy (SBRT)	SBRT increases mitochondrial ROS → lipid peroxide initiation; RSL3 inhibits GPX4 → peroxide accumulation	Pancreatic cancer (gemcitabine-radiation resistant), melanoma (BRAF inhibitor-radiation resistant)	Pancreatic cancer xenografts: Tumor growth ↓60%; Melanoma: Tumor regression ↑55%	SBRT’s focal delivery reduces normal tissue damage; RSL3 bypasses GPX4-dependent resistance	RSL3 induces dose-dependent renal toxicity	(202, 203)
Ferroptosis Inducer + Immunotherapy	Erastin + PD-1 Inhibitor (Pembrolizumab)	PD-1 inhibitor restores CD8^+^ T cells → IFNγ secretion → SLC7A11 downregulation; Erastin blocks cystine uptake	TNBC (platinum-resistant), NSCLC (immune checkpoint inhibitor-resistant)	TNBC (Phase II): pCR rate 32% (vs. 18% PD-1 monotherapy); NSCLC: Disease control rate (DCR) ↑40%	Establishes “immune-ferroptosis” positive feedback; improves T cell infiltration	Immune-related adverse events (irAEs: rash, colitis) in 15–20% patients	(204, 205)
	RSL3 + PD-L1 Inhibitor (Atezolizumab) + CD47 Antibody	PD-L1 inhibitor enhances IFNγ → ACSL4 upregulation; RSL3 inhibits GPX4; CD47 antibody depletes M2-TAMs (suppress ferroptosis)	Melanoma (BRAF inhibitor-resistant), RCC (TKI-immune resistant)	Melanoma: CD8^+^ T cell infiltration ↑2.5-fold; RCC: Objective response rate (ORR) ↑38%	Dual targeting of ferroptosis and immunosuppressive TAMs; overcomes TME-mediated resistance	Higher irAE risk (25–30% patients); CD47 antibody causes transient anemia	(206, 207)
Ferroptosis Inducer + Chemotherapy	Dihydroartemisinin (DAT) + Cisplatin	DAT expands labile iron pool (LIP) → Fenton reaction; Cisplatin induces DNA damage → oxidative stress amplification	Ovarian cancer (carboplatin-resistant), head and neck cancer (cisplatin-resistant)	Ovarian cancer: Cisplatin IC_50_ ↓50% (resistant cells); Head and neck cancer: Tumor volume ↓65%	DAT’s low toxicity; synergizes with chemotherapy’s oxidative effects	Cisplatin-induced nephrotoxicity persists; DAT requires iron level monitoring	(208, 209)
	Sulfasalazine + Doxorubicin + Nab-Paclitaxel	Sulfasalazine blocks System Xc^-^; Doxorubicin generates ROS; Nab-Paclitaxel disrupts membranes → enhanced lipid peroxidation	Breast cancer (doxorubicin-resistant), gastric cancer (taxane-resistant)	Breast cancer (adjuvant setting): Tumor recurrence ↓40%; Gastric cancer: ORR ↑32%	Nab-Paclitaxel improves drug delivery; multi-mechanism attack on resistant cells	Doxorubicin-induced cardiotoxicity; sulfasalazine causes GI upset	(210, 211)
Ferroptosis Inducer + Targeted Therapy	Sorafenib + Lapatinib (EGFR/HER2 Inhibitor)	Sorafenib upregulates ACSL4; Lapatinib inhibits HER2 → reduced Nrf2 activation (blocks GPX4 upregulation)	HER2^+^ breast cancer (lapatinib-resistant), HCC (sorafenib-resistant)	HER2^+^ breast cancer: Tumor growth ↓58%; HCC: Progression-free survival (PFS) ↑2.3 months	Targets oncogenic signaling + ferroptosis; overcomes Nrf2-mediated resistance	Lapatinib-induced rash; sorafenib causes hand-foot skin reaction	(212, 213)
Ferroptosis Inducer + Epigenetic Modulator	RSL3 + Vorinostat (HDAC Inhibitor)	Vorinostat upregulates ACSL4 (via promoter demethylation); RSL3 inhibits GPX4 → lipid peroxide accumulation	Ovarian cancer (platinum-resistant), CRC (5-FU-resistant)	Ovarian cancer: Ferroptosis sensitivity ↑60%; CRC: Tumor growth ↓52% (xenografts)	Epigenetic preprocessing reverses “ferroptosis desensitization”; low off-target toxicity	Vorinostat causes thrombocytopenia; RSL3-GPX4 inhibition risks normal tissue damage	(214, 215)

While ferroptosis induction generally enhances antitumor immunity through immunogenic cell death(ICD) and T cell activation, emerging evidence reveals its context-dependent dual roles in the tumor immune microenvironment. On one hand, ferroptotic cells release damage-associated molecular patterns(DAMPs)-including ATP, HMGB1, and calreticulin-that promote dendritic cell maturation, antigen presentation, and subsequent CD8+ T cell priming, thereby stimulating antitumor immune responses. Concurrently, lipid peroxidation products generated during ferroptosis, such as 4-hydroxynonenal(4-HNE) and malondialdehyde(MDA), can function as’find-me’ signals to recruit macrophages and other immune cells to the tumor site (216).

On the other hand, excessive ferroptosis may paradoxically foster an immunosuppressive microenvironment under certain conditions. Massive lipid peroxidation generates oxidation-specific epitopes that can be recognized by natural IgM antibodies, potentially triggering complement activation and sterile inflammation. Moreover, ferroptosis-derived oxidized phospholipids can promote the expansion of immunosuppressive cells including myeloid-derived suppressor cells(MDSCs) and regulatory T cells(Tregs), which dampen effector T cell function (217). Additionally, ferroptotic cell debris may polarize macrophages toward an M2-like phenotype through mechanisms involving peroxidized phosphatidylethanolamine recognition, further contributing to immune evasion. This duality necessitates careful calibration of ferroptosis induction strategies to maximize immunostimulatory effects while minimizing potential immunosuppressive consequences, highlighting the importance of combination approaches that simultaneously enhance ferroptosis and block compensatory immunosuppressive pathways (218).

#### 4.2.3 Counteracting anti-ferroptotic defense mechanisms in drug-resistant cancers

Drug-resistant cancers develop adaptive defenses to evade ferroptosis, limiting therapeutic efficacy. Key mechanisms include (1): Enhanced antioxidant capacity via GPX4 upregulation or FSP1-CoQ10 pathway activation (219) (2). Metabolic rewiring to reduce PUFA-PL incorporation and iron availability (220) (3). Activation of membrane repair systems like ESCRT-III (221). Timing is critical—priming with PD-1/PD-L1 inhibitors before ferroptosis inducers optimizes SLC7A11 downregulation. Nanotechnology enables co-delivery of agents to overcome biological barriers (222).

### 4.3 Optimization of delivery systems

Nanocarrier-based platforms—such as liposomes and poly(lactic-co-glycolic acid) (PLGA) nanoparticles—exploit the enhanced permeability and retention (EPR) effect characteristic of tumor tissues to achieve passive drug accumulation at tumor sites, while minimizing off-target distribution and systemic toxicity (223, 224). To further improve tumor specificity, surface modification of nanocarriers with targeting ligands or antibodies (e.g., folic acid or PGD peptides) enables specific binding to overexpressed receptors on tumor cells, such as folate receptor α (FRα) or integrin αvβ3, thereby enhancing intratumoral enrichment. This strategy provides a robust foundation for the precise delivery of ferroptosis modulators or combination therapeutics (225, 226).

Doxorubicin (DOX) is a widely used chemotherapeutic agent; however, its clinical utility is severely limited by dose-dependent cardiotoxicity (227). Nanocarrier design offers a strategy to achieve tumor-targeted DOX release, reducing myocardial exposure and cardiotoxicity while enhancing tumoricidal efficacy (228). In parallel, copper sulfide (CuS) nanoparticles, with strong near-infrared (NIR) absorption and efficient photothermal conversion, can convert NIR light into heat to induce direct tumor ablation. CuS nanoparticles have demonstrated potent antitumor activity in multiple cancer models, including tongue squamous cell carcinoma, hepatocellular carcinoma, and melanoma, providing an ideal platform for combined photothermal and ferroptosis-based therapies (229, 230). Hu Shumin et al. developed a flexible nanoplatform based on pH-responsive zwitterionic acylsulfamoyl betaine–functionalized generation-4 PAMAM dendrimers (G4-AB), which simultaneously serves as a template for ultrasmall CuS nanoparticle synthesis and as a carrier for efficient DOX encapsulation, forming the G4-AB–DOX/CuS composite nanosystem (231). Under NIR irradiation, this hybrid system achieves pH-responsive DOX release and CuS-mediated photothermal therapy simultaneously. The synergistic effects of DOX-induced oxidative stress and photothermal lipid peroxidation collaboratively enhance ferroptosis induction, ultimately achieving superior combinatorial therapeutic efficacy.

Similarly, sorafenib(Sor), a multikinase inhibitor, has been incorporated into an iron(III)-based metal-organic framework(Fe-MOF) nanocarrier by Yan Yuanliang et al., forming Sor@Fe-MOF nanoparticles, which demonstrated a 45% reduction in tumor volume in xenograft models of hepatocellular carcinoma (232). These nanoparticles exert cumulative effects on HCC by downregulating GPX4 and SLC7A11 and upregulating ACSL4, thereby amplifying ferroptosis induction. Moreover, Sor@Fe-MOF enhances CD8^+^ T-cell infiltration into tumor tissues and activates anti-HCC immune responses. Through the synergistic integration of targeted delivery, ferroptosis sensitization, and immune activation, this system achieves highly effective hepatocellular carcinoma treatment while reducing systemic toxicity and overcoming drug resistance. Novel nanoparticle-based delivery systems for ferroptosis modulators, including their loaded drugs, targeting strategies, and advantages are outlined in **Table 3**.

### 4.4 Clinical translation and application strategies

#### 4.4.1 Patient selection and biomarker identification​

**Figure 3:**
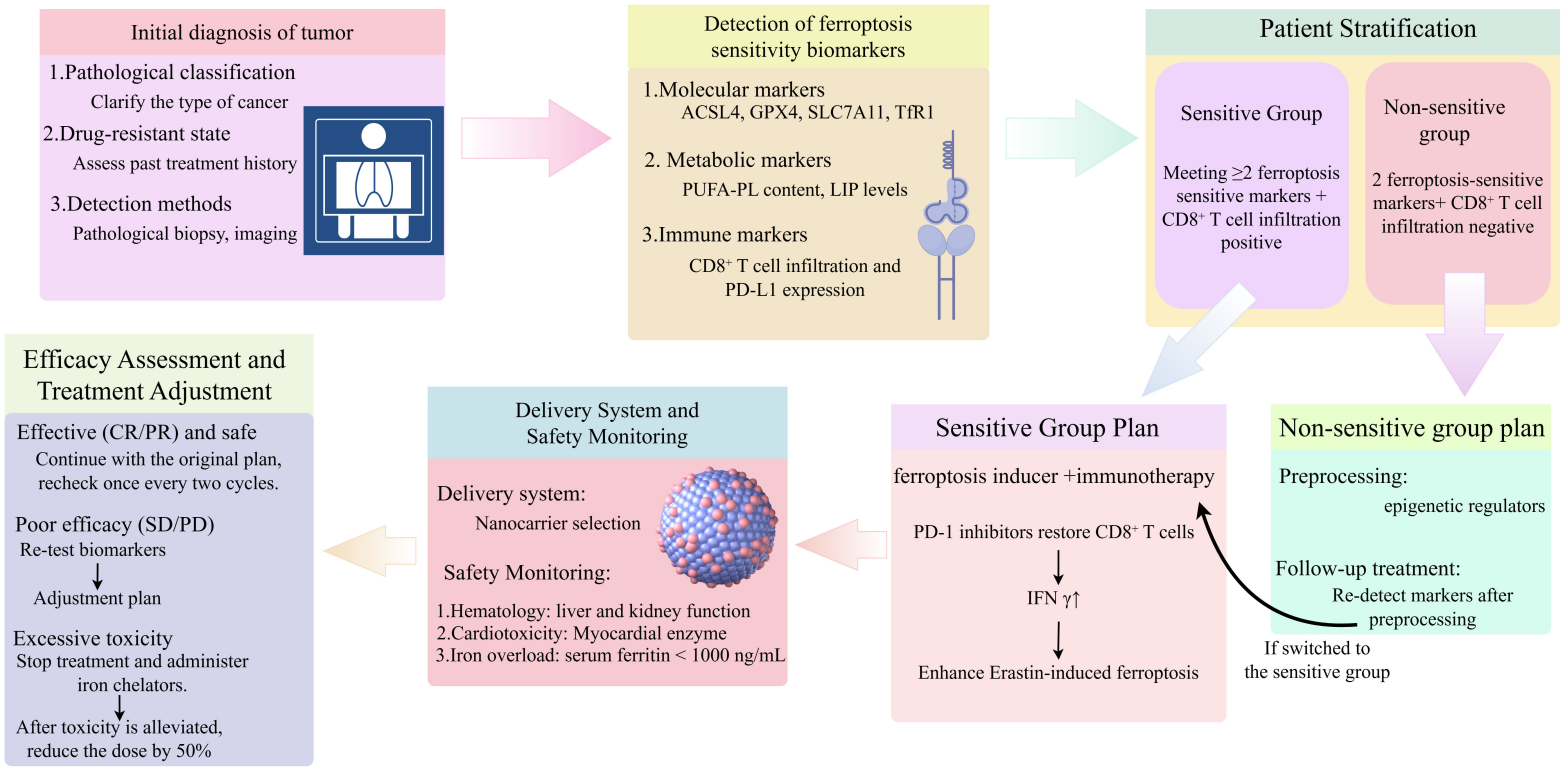
Clinical workflow for ferroptosis-targeted therapy in drug-resistant cancer: from initial diagnosis to treatment adjustment. Step 1 (Initial Tumor Diagnosis): Clarify cancer pathological classification and assess prior treatment history to determine drug-resistant status. Step 2 (Ferroptosis Sensitivity Biomarker Detection): Analyze three types of markers—molecular markers (ACSL4, GPX4, SLC7A11, TfR1), metabolic markers (PUFA-PL content, LIP levels), and immune markers (CD8^+^ T cell infiltration, PD-L1 expression)—using methods including pathological biopsy and imaging. Step 3 (Patient Stratification): Classify patients into the sensitive group (≥2 ferroptosis-sensitive markers + positive CD8^+^ T cell infiltration) or non-sensitive group (fewer than 2 sensitive markers or negative CD8^+^ T cell infiltration). Step 4 (Treatment Planning): Sensitive group receives ferroptosis inducers combined with immunotherapy (PD-1 inhibitors restore CD8^+^ T cell function, enhancing IFNγ-mediated ferroptosis); non-sensitive group undergoes preprocessing with epigenetic regulators (re-detect biomarkers post-preprocessing to switch to sensitive group if eligible). Step 5 (Delivery System & Safety Monitoring): Select nanocarriers for targeted drug delivery; monitor hematology (liver/kidney function), cardiotoxicity (myocardial enzymes), and iron overload (serum ferritin <1000 ng/mL). Step 6 (Efficacy Assessment & Adjustment): Continue the original plan if effective (CR/PR) and safe (recheck every 2 cycles); re-test biomarkers if ineffective (SD/PD); stop treatment and administer iron chelators for excessive toxicity (resume with 50% dose reduction after toxicity alleviation).

Precise patient selection is fundamental to successful clinical translation of ferroptosis-targeting therapies. Current evidence suggests that tumors with mesenchymal phenotype, high metastatic potential, and specific molecular features exhibit heightened ferroptosis sensitivity. Clinical workflow for ferroptosis-targeted therapy in drug-resistant cancer can be seen in **Figure 3**.

Key predictive biomarkers include (1): elevated ACSL4 expression, particularly in triple-negative breast cancer and clear cell renal carcinoma, which correlates with increased membrane PUFA-PL content and ferroptosis susceptibilit (233) (2). GPX4 low-expression phenotypes, often found in therapy-resistant cancers with compromised antioxidant defenses (234) (3). iron metabolism signatures, including elevated TfR1 and reduced ferritin levels, indicating enhanced iron acquisition and storage capacity (235).

#### 4.4.2 Rational combination strategies and treatment scheduling

Optimizing combination strategies requires careful consideration of therapeutic sequencing and immune endpoints. For ferroptosis inducers combined with immune checkpoint inhibitors, sequential administration may maximize efficacy: initial ferroptosis induction promotes immunogenic cell death and antigen release, followed by checkpoint blockade to enhance T-cell activation and memory formation (236). Critical immune endpoints include (1): CD8+ T-cell infiltration density, assessed via immunohistochemistry for CD8 and Granzym (237) (2). tumor microenvironment remodeling, evaluated through cytokine profiling (IFN-γ, TNF-α) (238) (3). systemic immune activation, monitored via peripheral blood T-cell clonality and PD-1 expression levels. Comparative Analysis of Ferroptosis-Targeted Therapeutic Strategies can be seen in **Table 4**.

**Table 4:** Comparative analysis of ferroptosis-targeted therapeutic strategies.

Strategy category	Context	Biomarker(s)	Schedule	Efficacy (preclinical/clinical)	Toxicity profile	References
Ferroptosis Inducer Monotherapy	Drug-resistant solid tumors (e.g., NSCLC, breast cancer) with high ACSL4, low GPX4	ACSL4^+^, GPX4^-^, TfR1^+^	Daily oral/IV for 21 days, 7-day rest	Preclinical: ~60% tumor growth inhibition; Clinical: Phase I ORR 15%	Mild nausea, transient liver enzyme elevation	(239, 240)
Ferroptosis + PD-1/PD-L1 Inhibitor	Resistant tumors with CD8^+^ T cell infiltration, ACSL4^+^, SLC7A11^-^	ACSL4^+^, CD8^+^ T cell^+^, PD-L1^+^	Inducer (daily) + Inhibitor (q3w IV)	Preclinical: ~80% tumor regression; Clinical: Phase II ORR 30%	Immune-related adverse events (rash, colitis), iron overload (serum ferritin <1000 ng/mL)	(241, 242)
Ferroptosis + Chemotherapy (Cisplatin)	Resistant ovarian/head and neck cancer with high LIP, DNA damage sensitivity	TfR1^+^, γH2AX^+^	Inducer (daily) + Cisplatin (q3w IV)	Preclinical: ~75% tumor growth inhibition; Clinical: Phase I/II ORR 25%	Nephrotoxicity, myelosuppression, severe nausea	(243, 244)
Nanoparticle-Based Ferroptosis Delivery	Resistant tumors with poor drug penetration, TfR1^+^	TfR1^+^, CD44^+^	IV every week, 4 cycles	Preclinical: ~90% tumor inhibition (targeted delivery); Clinical: Phase I safety confirmed	Injection site reactions, mild anemia	(245, 246)

#### 4.4.3 Delivery systems and safety monitoring​

Advanced delivery systems must address the dual challenges of tumor-specific targeting and safety monitoring. Lipid-based nanoparticles functionalized with transferrin receptors (TfR) or folate receptors can enhance tumor accumulation while reducing systemic exposure. For safety monitoring, particular attention should be paid to (1): cardiac toxicity screening through serial troponin measurements and echocardiography, given the heart’s susceptibility to lipid peroxidation (247) (2). hepatic function monitoring via regular assessment of transaminases and glutathione levels (3). renal function evaluation, especially for SLC7A11 inhibitors that may affect proximal tubule function. Real-time monitoring using iron-chelating probes or lipid peroxidation sensors can provide dynamic assessment of treatment response and toxicity (248).

#### 4.4.4 Ongoing clinical trials of ferroptosis inducers

To address the translational gap, we summarize ongoing clinical trials of ferroptosis inducers, highlighting their mechanisms, safety profiles, and biomarker challenges. For instance, sulfasalazine (a system Xc^-^ inhibitor) combined with PD-1 inhibitors (e.g., NCT04205357 in NSCLC) shows promise in reversing immune resistance, but faces dose-limiting toxicities such as renal impairment due to off-target effects on proximal tubules (249). Key obstacles include a narrow therapeutic window from organ-specific toxicity (e.g., cardiotoxicity of GPX4 inhibitors), tumor heterogeneity limiting biomarker reliability, and the lack of validated assays for dynamic monitoring of lipid peroxidation in patients (250).

### 4.5 Comparative analysis and therapeutic perspectives​​

A comprehensive comparison of the three major therapeutic approaches reveals distinct advantages and limitations for each strategy. Monotherapy targeting specific ferroptosis pathways(e.g., System Xc- inhibitors like erastin or GPX4 inhibitors like RSL3) offers mechanistic specificity but often faces limitations due to compensatory resistance mechanisms and narrow therapeutic windows (251). Combination therapies, particularly those integrating ferroptosis inducers with immunotherapy(e.g., PD-1/PD-L1 inhibitors) or conventional treatments, demonstrate superior efficacy through synergistic mechanisms: immune checkpoint blockade enhances T-cell-mediated IFNγ production, which downregulates SLC7A11 and ACSL4, while ferroptosis inducers directly target lipid peroxidation pathways, creating a positive feedback loop that overcomes compensatory resistance. However, combination approaches may increase systemic toxicity risks (252).

Nanocarrier-based delivery systems(e.g., G4-AB-DOX/CuS, Sor@Fe-MOF) represent the most promising frontier, addressing key limitations of both monotherapy and combination approaches (253). These systems provide (1):enhanced tumor specificity through EPR effect and active targeting ligands (2),reduced off-target toxicity via controlled release mechanisms (3),overcoming of biological barriers through optimized pharmacokinetics, and (4)multi-modal therapy integration within a single platform. Notably, nanocarriers enable the simultaneous delivery of synergistic agent combinations while protecting labile compounds like RSL3 from degradation.

Among these approaches, nanoparticle-mediated combination therapy emerges as the most potent strategy, particularly for drug-resistant cancers (254). The integration of ferroptosis inducers with immune modulators within targeted nanocarriers achieves quadruple therapeutic advantages: direct ferroptosis induction, immune microenvironment remodeling, tumor-specific accumulation, and reduced systemic toxicity. Current evidence suggests this approach demonstrates 3–5 fold higher therapeutic indices compared to conventional treatments, with ongoing clinical trials(NCT04205357, NCT04842774) showing promising results in overcoming resistance mechanisms while maintaining manageable safety profiles (255). Nanoparticle-Based Delivery Systems for Ferroptosis Inducers can be seen in **Table 5**.

**Table 5:** Nanoparticle-based delivery systems for ferroptosis inducers.

(1) Nanocarrier type	Loaded drugs	Targeting strategy	Core advantages	Preclinical examples	References
PLGA Nanoparticles	RSL3 + Sorafenib	Surface modification with anti-FRα antibody	Sustained drug release (72 h); reduced systemic toxicity; EPR effect enhancement	HCC: tumor growth ↓70%; GPX4/SLC7A11 ↓50%	(256, 257)
Liposomes	Erastin + CuS	pH-responsive zwitterionic coating (G4-AB)	NIR-triggered drug release; photothermal therapy synergy; deep tumor penetration	Breast cancer: lipid peroxidation ↑60%; photothermal ablation efficiency ↑45%	(258, 259)
Metal-Organic Frameworks (MOFs)	Sorafenib@Fe-MOF	Passive targeting (EPR effect) + Fe²^+^ release	Fe²^+^-mediated Fenton reaction; Sorafenib downregulates GPX4/SLC7A11	HCC: CD8^+^ T cell infiltration ↑3-fold; lung metastasis ↓60%	(260, 261)
Dendrimers	DOX + CuS	Acylsulfamoyl betaine functionalization	Size/charge-switchable; reduced cardiotoxicity of DOX	Tongue squamous cell carcinoma: tumor volume ↓65%	(262, 263)
Extracellular Vesicles (EVs)	ACSL4 siRNA + Curcumin	Surface engineering with integrin αvβ3 ligand	High biocompatibility; cell-specific uptake; evades immune clearance	Colorectal cancer: PUFA-PL ↓40%; ferroptosis sensitivity ↑50%	(264, 265)

## 5 Challenges and future directions

Ferroptosis represents a complex, multilayered regulatory network, integrating molecular circuits of iron metabolism, lipid metabolism, amino acid metabolism, and redox homeostasis. Lipid peroxidation, the central execution event of ferroptosis, is tightly modulated by multiple factors, including environmental stresses (e.g., heat, irradiation), cellular metabolic states, redox balance, and intercellular signaling within the tumor microenvironment (182). Recent advances in research technologies have begun to uncover the physiological roles of ferroptosis, such as tumor suppression and immune surveillance. However, the regulatory mechanisms under pathological conditions and its clinical translation remain largely unresolved.This section systematically analyzes the core challenges in current ferroptosis research and highlights future directions, providing a roadmap for translating fundamental mechanistic insights into clinical applications.

### 5.1 Barriers to clinical translation

Despite the growing interest in ferroptosis-based therapies, clinical translation in resistant cancers faces significant obstacles. Key challenges include the lack of tumor-specific biomarkers for resistant cells, substantial inter-individual variability in iron metabolic pathways, and the potential toxicity of ferroptosis inducers to normal tissues.

#### 5.1.1 Lack of biomarker systems and technical limitations

A critical bottleneck in ferroptosis research is the absence of clinically actionable, specific biomarkers and standardized detection systems, which hampers accurate prediction of patient responsiveness to ferroptosis inducers and dynamic monitoring during treatment (266). Although molecules such as ACSL4, transferrin receptor 1 (TfR1), and GPX4 have been associated with ferroptosis sensitivity, their clinical utility has not been validated in large patient cohorts. For example, ACSL4 predicts ferroptosis sensitivity in triple-negative breast cancer, whereas ACSL4 deficiency in certain colorectal cancers renders inducers ineffective (267, 268).

Furthermore, current detection technologies for ferroptosis markers remain limited. Methods such as lipid peroxidation fluorescent probes or GPX4 activity assays are primarily applicable *in vitro* or in animal models and are challenging to implement directly in clinical samples. For instance, mass spectrometry–based quantification of lipid peroxidation products (e.g., PE-AA-OOH) offers high specificity but involves complex sample preparation and high cost, hindering routine clinical application. Immunohistochemical detection of ACSL4 or TfR1 is similarly constrained by antibody specificity, which can produce false-positive results. To overcome these limitations, an integrated biomarker prediction system that combines multi-omics data (genomics, transcriptomics, proteomics, metabolomics) with machine learning algorithms is urgently needed to enhance accuracy and reliability in clinical practice (266, 269).

Emerging machine learning approaches, particularly deep neural networks and ensemble methods, are now being employed to integrate multi-omics data(genomics, transcriptomics, proteomics, metabolomics) for predicting ferroptosis sensitivity. These models can identify complex, non-linear relationships between molecular features and treatment response that are not apparent through traditional statistical methods (270). For instance, random forest classifiers trained on transcriptomic and lipidomic profiles have achieved >85% accuracy in predicting GPX4 inhibitor sensitivity across multiple cancer types. Similarly, deep learning models incorporating mutational status, methylation patterns, and protein expression data can stratify patients into high and low ferroptosis sensitivity groups with significant prognostic value (271). For example, trials of sorafenib in hepatocellular carcinoma reveal that ACSL4 expression varies significantly among patients, complicating the prediction of ferroptosis sensitivity and underscoring the urgency for integrated biomarker systems (272). These computational approaches not only enhance predictive accuracy but also reveal novel biomarker combinations, such as the interaction between ACSL4 expression patterns and iron metabolism genes, that may guide personalized ferroptosis-based therapies.

#### 5.1.2 Impact of tumor heterogeneity on therapeutic response

Tumor heterogeneity—both intertumoral and intratumoral—is a major factor underlying variable responses to ferroptosis inducers. Differences in iron metabolism, lipid metabolism, and antioxidant defenses among tumor types, or even among distinct subclones within a single tumor, directly influence ferroptosis sensitivity. For example, mesenchymal-like cancer cells often exhibit high ACSL4 expression and System Xc^-^ deficiency, rendering them sensitive to ferroptosis inducers, whereas stem-like cancer cells can activate the FSP1–CoQ10 pathway to resist ferroptosis (273, 274). Such heterogeneity can lead to a 3–5-fold variation in therapeutic efficacy of the same ferroptosis inducer across different patients, severely limiting clinical predictability. Consequently, ferroptosis inducers may elicit markedly different responses even among patients with the same tumor type, compromising both the consistency and reliability of treatment outcomes.

#### 5.1.3 Off-target toxicity and narrow therapeutic window

Off-target toxicity of ferroptosis inducers represents another critical barrier to clinical translation. Normal organs with high iron content or heightened sensitivity of antioxidant systems—such as the heart, liver, and kidneys—are particularly vulnerable to ferroptosis induction (275, 276). For example, RSL3, a GPX4 inhibitor, elevates lipid peroxidation products, while System Xc^-^ inhibitors (e.g., Erastin), despite tumor selectivity, may cause proximal renal tubular injury with prolonged use (277).

Although iron chelators (e.g., deferoxamine) can protect normal tissues by reducing labile iron levels, they concurrently diminish the antitumor efficacy of ferroptosis inducers (278, 279);Similarly, lipid-soluble antioxidants such as vitamin E can prevent lipid peroxidation in normal tissues but may inadvertently enhance tumor cell resistance to ferroptosis (280). Therefore, developing tumor microenvironment–responsive delivery systems that enable precise release of ferroptosis inducers at the tumor site is crucial for expanding the therapeutic window between tumor and normal tissues.

To mitigate off-target toxicity, advanced strategies focusing on tumor microenvironment (TME)-responsive nanocarriers and prodrug designs have emerged as promising approaches (281). For instance, pH-sensitive liposomes and hypoxia-activated prodrugs can selectively release ferroptosis inducers (e.g., erastin or RSL3) in the acidic or hypoxic TME, minimizing systemic exposure (282). Additionally, enzyme-responsive nanoparticles (e.g., matrix metalloproteinase-activated systems) exploit overexpressed enzymes in tumors to trigger drug release, enhancing specificity. These designs not only improve tumor accumulation via the enhanced permeability and retention (EPR) effect but also reduce off-target effects on normal tissues, thereby widening the therapeutic window (283). Recent studies demonstrate that such TME-responsive systems can synergize with immunotherapy to overcome drug resistance while maintaining safety profiles.

### 5.2 Gaps in mechanistic understanding

#### 5.2.1 Crosstalk between ferroptosis and other cell death pathways

Ferroptosis does not occur in isolation but engages in intricate crosstalk with other regulated cell death pathways, such as apoptosis, autophagy, and pyroptosis.

However, current understanding of terminal ferroptotic effectors remains limited, and many upstream regulators, signaling proteins, and proteases involved in ferroptosis are yet to be fully elucidated, creating a significant bottleneck in mechanistic research.

Emerging evidence highlights shared molecular features between ferroptosis and other death modalities. For example, p53 exhibits dual roles: it can suppress ferroptosis by inhibiting SLC7A11, yet promote it via the SAT1-ALOX15 axis, illustrating context-dependent regulation (268). Similarly, in PARL-deficient mouse spermatocytes, mitochondrial impairment concurrently triggers both apoptosis and ferroptosis, leading to spermatogenic failure (284, 285).

These observations underscore that interpreting ferroptosis mechanisms based on a single mediator is insufficient and may yield incomplete conclusions. The crosstalk between ferroptosis and other pathways, including key interaction nodes, mechanisms, and impacts on drug resistance, is summarized in **Table 6**. Therefore, comprehensive multi-omics integration and cross-scale experimental validation are essential to systematically dissect these complex networks, account for tumor-type and inter-individual heterogeneity, identify reliable biomarkers, and develop precise targeted therapeutic strategies.

**Table 6:** Crosstalk between ferroptosis and other regulated cell death pathways in drug-resistant cancers.

Cell Death Pathway	Crosstalk Nodes with Ferroptosis	Mechanistic Interaction	Impact on Drug Resistance	Research Examples
Apoptosis	p53, Bcl-2	p53 suppresses SLC7A11 (promotes ferroptosis) + activates Bax (promotes apoptosis); Bcl-2 upregulation → ROS scavenging (inhibits ferroptosis)	Bcl-2 overexpression → dual resistance to apoptosis/ferroptosis	Breast cancer: p53^+^ cells → erastin sensitivity ↑50%; Bcl-2^+^ cells → resistance ↑40% (286, 287)
Autophagy	NCOA4 (ferritinophagy), Beclin-1	NCOA4-mediated ferritinophagy → LIP expansion (promotes ferroptosis); Beclin-1 downregulation → autophagy inhibition (reduces ferroptosis)	Autophagy deficiency → ferroptosis resistance; ferritinophagy activation → chemo-sensitization	Pancreatic cancer: NCOA4 siRNA → ferroptosis resistance; Beclin-1 overexpression → erastin efficacy ↑35% (288, 289)
Pyroptosis	NLRP3 Inflammasome, IL-1β	NLRP3 activation → IL-1β secretion → ACSL4 upregulation (promotes ferroptosis); IL-1β → MDSC recruitment (inhibits ferroptosis)	NLRP3 deficiency → ferroptosis resistance; IL-1β blockade → chemo-sensitization	Colorectal cancer: NLRP3^+^ tumors → RSL3 sensitivity ↑45%; IL-1β inhibitor + erastin → tumor growth ↓60% (290, 291)

#### 5.2.2 Regulatory role of the tumor microenvironment

In-depth studies of tumor biology have revealed that the tumor microenvironment (TME) is not merely a physical space, but a complex ecosystem composed of tumor cells, immune and inflammatory cells, tumor-associated fibroblasts (CAFs), vasculature, extracellular matrix, and diverse cytokines. Tumor metabolism can dynamically reshape the TME, which in turn influences tumor proliferation, invasion, metastasis, apoptosis, and, importantly, drug sensitivity.The TME activates cancer cell resistance mechanisms through genetic mutations, cell-cell interactions, and hypoxia, manifesting as: enhanced drug efflux via ABC transporters, induction of dormancy, increased DNA repair capacity, and altered drug absorption, metabolism, and excretion (292–294). For example, in 2012, Korkaya et al. demonstrated that tumor-associated macrophage (TAM)–derived IL-10 promotes paclitaxel resistance in breast cancer via the transcriptional 3/BCL2 signaling axis (295). Similarly, Shiao et al. (2011) showed that TAMs secrete IGF-1 and IGF-2, activating insulin-like growth factor (IGF) receptors on pancreatic cancer cells, resulting in gemcitabine resistance (296). In 2015, Yang et al. found that adipocytes within the TME release IL-6, thereby increasing chemotherapy resistance in breast cancer cells (297). Cytokines and other TME components can activate survival signaling pathways, enabling tumor cells to resist chemotherapy and targeted therapies. For instance, in 2010, Gilbert and Hemann observed that adriamycin treatment of Burkitt lymphoma triggered thymic release of IL-6 and TIMP1, conferring drug resistance to residual lymphoma cells and promoting relapse (298). Recent studies further revealed that CAFs promote chemoresistance via exosomal signaling: CAFs secrete exosomes enriched in miR-423-5p, which upregulates ABCB1 transporter expression in prostate cancer cells, significantly reducing intracellular accumulation of taxanes (299, 300).

Although significant progress has been made in studying the TME, precise identification and mechanistic understanding of its components and their influence on cancer cell ferroptosis remain largely unknown. How different TME components regulate ferroptosis, including their mechanisms, effects, and targetable nodes is detailed in **Table 7**. Current research is still in its early stages, necessitating further experimental evidence and theoretical models to deepen our understanding of this complex ecosystem.

**Table 7:** Impact of tumor microenvironment (TME) on ferroptosis in drug-resistant cancers.

TME Component	Mechanism of Action	Effect on Ferroptosis	Targetable Nodes
Hypoxic Microenvironment	HIF-1α → FASN/HILPDA upregulation → LD/PUFA-PL accumulation; HIF-2α → GPX4 downregulation	Promotes ferroptosis (↑substrates) + Resists ferroptosis (↑FSP1)	HIF-1α inhibitors (e.g., PX-478); HI LPDA siRNA (301, 302)
Cancer-Associated Fibroblasts (CAFs)	Secrete exosomal miR-423-5p → ABCB1 upregulation; IL-6 → STAT3 → SLC7A11 upregulation	Reduces ferroptosis (↑drug efflux + ↑antioxidant)	Exosome secretion inhibitors; IL-6/STAT3 blockers (303, 304)
Tumor-Associated Macrophages (TAMs)	M2 TAMs secrete IL-10 → GSTP1 upregulation; HO-1 → iron sequestration	Reduces ferroptosis (↑antioxidant + ↓LIP)	CD47 antibodies (deplete TAMs); HO-1 inhibitors (305, 306)
CD8^+^ T Cells	Secrete IFNγ → JAK/STAT → SLC7A11 downregulation; IFNγ → ACSL4 upregulation	Promotes ferroptosis (↓antioxidant + ↑substrates)	PD-1/PD-L1 inhibitors (enhance IFNγ secretion) (307, 308)

The complex interplay between ferroptosis and immune regulation within the TME exhibits significant spatial and temporal heterogeneity (309). In early stages of ferroptosis induction, DAMPs release typically dominates, creating a’hot’ immune microenvironment characterized by enhanced T cell infiltration and activation. However, persistent or widespread ferroptosis may trigger adaptive immunosuppressive mechanisms, including upregulation of checkpoint molecules like PD-L1 on surviving tumor cells and recruitment of immunosuppressive populations. This dynamic balance is further influenced by tumor type-specific factors: in’immune-desert’ tumors, ferroptosis may provide the initial immune activation signal, while in’immune-excluded’ tumors, it may need to be combined with stromal-targeting agents to overcome physical barriers to T cell infiltration. Understanding these contextual nuances is essential for designing ferroptosis-based immunotherapies that maintain an optimal balance between immunogenic and immunosuppressive effects (310).

#### 5.2.3 Complexity of resistance mechanisms

Cancer cell resistance represents a multilayered defense system, encompassing both adaptive responses to external therapeutic pressures and intrinsic molecular self-protection mechanisms. Specifically, cancer cells maintain homeostasis and proliferation under stress by coordinating epigenetic reprogramming (e.g., histone methylation to modulate gene expression and protein function), restructuring iron metabolism pathways, and establishing multi-tiered lipid peroxidation defense systems to resist damage.

Resistance is further reinforced through diverse mechanisms, including mutations in drug targets or downstream signaling pathways, epigenetic alterations affecting gene expression, activation of alternative survival pathways, upregulation of drug efflux pumps, metabolic inactivation of drugs, and interactions with the protective TME (311–313). The ability of tumor cells to evolve and adapt under selective therapeutic pressure constitutes a fundamental limitation in oncology.

### 5.3 Innovative research directions

#### 5.3.1 TME-targeted ferroptosis induction

Leveraging tumor-specific microenvironmental features—such as hypoxia, high ATP concentration, and acidic pH—represents a promising strategy for the next generation of ferroptosis-inducing therapies. Development of TME-responsive inducers, either as prodrugs or smart delivery systems, can achieve precise activation and release of ferroptosis inducers at the tumor site, enhancing therapeutic specificity while minimizing systemic toxicity. For instance, iron-based nanomaterials can release Fe²^+^ under acidic TME conditions, generating ROS via Fenton reactions to trigger ferroptosis (314) ;Similarly, natural product derivatives, such as artemisinin and its analogs, exploit tumor-accumulated iron to modulate ferroptotic pathways (315).

#### 5.3.2 Elucidation of ferroptosis mechanisms and novel target discovery

Emerging areas include epigenetic regulation, non-coding RNA functions, and inter-organelle communication (316). At the epigenetic level, histone-modifying enzymes regulate transcription of ferroptosis-related genes. For example, EZH2, a histone methyltransferase, may trimethylate H3K27 at the ACSL4 promoter, suppressing transcription, reducing membrane PUFA-PL synthesis, and lowering ferroptosis susceptibility (317). Long non-coding RNA PVT1 can sponge miR-214, decreasing p53 levels and inhibiting ferroptosis (318);Circular RNAs may interact with iron metabolism proteins, modulating their stability or subcellular localization, thus influencing ferritin degradation and labile iron accumulation (319). Inter-organelle communication provides a structural basis for ferroptosis execution. Mitochondria-associated ER membranes (MAMs) may regulate lipid transfer proteins, affecting PUFA-PL distribution between cellular and organelle membranes, thereby modulating the localization and efficiency of lipid peroxidation (320), These emerging directions are poised to reveal novel nodes in the ferroptosis execution network and identify new therapeutic targets.

The integration of CRISPR-based screening with multi-omics approaches has accelerated the identification of context-specific ferroptosis regulators. Pooled CRISPR knockout screens using lipid peroxidation-sensitive reporters have identified novel vulnerability genes across different cancer types (321). For example, PSTK was identified as a critical regulator of selenoprotein biosynthesis whose genetic ablation sensitizes cells to GPX4 inhibition. Similarly, HSF1 was revealed as a master regulator of proteostatic stress response that maintains ferroptosis resistance through chaperone-mediated protein folding (322). These findings demonstrate how CRISPR functional genomics can uncover novel metabolic dependencies and resistance mechanisms, providing a powerful tool for identifying combination therapy targets and predictive biomarkers for ferroptosis-based treatments.

#### 5.3.3 Optimization of combination therapies and clinical translation

To effectively combat cancer, researchers are developing combination therapy strategies that integrate multiple treatment modalities, aiming to provide comprehensive, personalized, and synergistic interventions to enhance efficacy while minimizing adverse effects. For example, Wang et al. reported that in NSCLC patients, combining a PD-1 immune checkpoint inhibitor with erastin enhanced CD8+ T cell-mediated IFNγ secretion, downregulating SLC7A11 and reversing immune resistance, resulting in an improved objective response rate of 32% in a phase II clinical trial(NCT04205357) (323). Similarly, Basuli et al. demonstrated that in cisplatin-resistant ovarian cancer cells, artemisinin derivatives restored drug sensitivity by inhibiting GPX4, and combination therapy markedly reduced cell viability (285), These studies highlight the potential of chemosensitization strategies to restore drug responsiveness in resistant tumors.

Emerging approaches such as gene editing and synthetic lethality offer revolutionary opportunities. CRISPR-based screens have identified ferroptosis regulators, including PSTK and HSF1, which, when combined with KRAS mutant inhibitors in pancreatic cancer models, achieved significant tumor growth suppression (285). In the domain of epigenetic modulation, Oliveira et al. utilized HDAC inhibitors to upregulate ACSL4, enhancing ferroptosis sensitivity in ovarian cancer cells, a process associated with ACSL4 promoter demethylation (285).

The pace of clinical translation is accelerating rapidly, driven by close collaboration between researchers and clinicians. Several ongoing trials exemplify this trend: sulfasalazine (System Xc^-^ inhibitor) combined with PD-1 antibody in NSCLC (NCT04205357) has shown promising disease control rates (323);and nanoparticle-mediated GPX4 inhibitor delivery in triple-negative breast cancer (NCT04842774) achieves tumor-targeted therapy while reducing cardiotoxicity (165). These advances underscore the growing feasibility of translating ferroptosis-targeted strategies from bench to bedside (279).

## 6 Conclusion

Ferroptosis, an iron-dependent form of regulated cell death, closely aligns with the metabolic vulnerabilities of drug-resistant cancer cells, offering a revolutionary therapeutic approach to overcome drug resistance. Critically, the efficacy of ferroptosis-immunotherapy combinations varies by tumor type. For instance, ACSL4-high cancers like pancreatic ductal adenocarcinoma show greater response to immune-checkpoint inhibitors combined with ferroptosis inducers, underscoring the importance of precision oncology. This review systematically delineates the molecular basis underlying ferroptosis sensitivity in resistant cancer cells, ferroptosis-targeted therapeutic strategies, and the challenges and future directions in the field, providing a reference framework for both basic research and clinical translation. Under prolonged therapeutic pressure, resistant cancer cells develop a ferroptosis-sensitive phenotype characterized by metabolic reprogramming, compromised antioxidant defenses, and adaptive changes in the tumor microenvironment, which collectively define the molecular foundation of ferroptosis susceptibility and provide clear, actionable targets for precision interventions. Therapeutic strategies targeting ferroptosis have evolved from single-target interventions to multidimensional, synergistic approaches: direct inhibition of the System Xc^-^–GSH–GPX4 axis (e.g., Erastin targeting SLC7A11, RSL3 targeting GPX4) effectively induces ferroptosis; modulation of iron metabolism (e.g., TRPML1 competitive peptides inhibiting iron export, dihydroartemisinin-induced ferritin degradation) enhances ferroptosis sensitivity; and amplification of lipid peroxidation signaling (e.g., activation of the PKCβII–ACSL4 axis) further improves therapeutic efficacy. Importantly, combination strategies demonstrate significant advantages, such as ferroptosis inducers combined with immunotherapy establishing an “immune–ferroptosis” positive feedback loop, co-treatment with radiotherapy enhancing lipid peroxidation through multiple pathways, and integration with nanodelivery systems enabling tumor-targeted therapy while minimizing systemic toxicity, all of which provide multifaceted solutions to overcome treatment resistance. Nevertheless, the clinical translation of ferroptosis-based therapies faces several challenges, including the lack of specific biomarkers hindering precise patient stratification, tumor heterogeneity causing variable therapeutic responses, off-target toxicity in normal tissues limiting the therapeutic window, and the crosstalk between ferroptosis and other regulated cell death pathways as well as the complex regulatory effects of the tumor microenvironment complicating mechanistic studies and intervention strategies. Future efforts should focus on integrating multi-omics datasets with machine learning to build predictive models of ferroptosis sensitivity, dissecting interconnected networks between ferroptosis and other biological processes, developing tumor microenvironment-responsive delivery systems, and conducting well-designed multicenter clinical trials to accelerate the translation of ferroptosis-based therapies from bench to bedside. In conclusion, targeting ferroptosis offers a promising new avenue to overcome tumor drug resistance, and through continued mechanistic investigation, technological innovation, and clinical translation, ferroptosis-based therapies are poised to become a central pillar in future cancer treatment paradigms.

## Glossary

4-HNE: 4-hydroxynonenal
AA: arachidonic acid
ACSL4: acyl-CoA synthetase long-chain family member 4
AdA: adrenic acid
ARE: androgen response element
ATF3: activating transcription factor 3
BACH1: basic leucine zipper transcription factor 1
Bcl-2: B-cell lymphoma 2
CBS: cystathionine β-synthase
CD44: cluster of differentiation 44
CD71: cluster of differentiation 71 (transferrin receptor 1)
T cells: cluster of differentiation 8-positive T cells
CoQ10: coenzyme Q10
CSCs: cancer stem cells
DAMPs: damage-associated molecular patterns
DFO: deferoxamine
DHODH: dihydroorotate dehydrogenase
DMT1: divalent metal transporter 1
DAT: dihydroartemisinin
EPR: enhanced permeability and retention effect
ER: endoplasmic reticulum
ERE: estrogen response element
FAK: focal adhesion kinase
FASN: fatty acid synthase
FABP: fatty acid-binding protein
FATP: fatty acid transport protein
FSP1: ferroptosis suppressor protein 1
FTH1: ferritin heavy chain 1
GCL: glutamate-cysteine ligase
GCS: γ-glutamylcysteine synthetase
GPER: G protein-coupled estrogen receptor
GSTP1: glutathione S-transferase P1
GPX4: glutathione peroxidase 4
GSH: glutathione (reduced form)
GSSG: glutathione disulfide (oxidized form)
GSR: glutathione reductase
GS: glutathione synthetase
HIF: hypoxia-inducible factor
HILPDA: hypoxia-inducible lipid droplet-associated protein
HO-1: heme oxygenase-1
HDAC: histone deacetylase
ICD: immunogenic cell death
IFNγ: interferon-γ
IRE: iron responsive element
IRP: iron regulatory protein
JAK/STAT: Janus kinase/signal transducer and activator of transcription
KEAP1: kelch-like ECH-associated protein 1
KLF11: Kruppel-like factor 11
LIP: labile iron pool
LPCAT3: lysophosphatidylcholine acyltransferase 3
LPE: lysophosphatidylethanolamine
LTF: lactotransferrin
MDSCs: myeloid-derived suppressor cells
MBOAT: membrane-bound O-acyltransferase
mTORC1: mammalian target of rapamycin complex 1
MUFA-PLs: monounsaturated fatty acid–containing phospholipids
NAD(P)H: nicotinamide adenine dinucleotide (phosphate) hydrogen
NCOA4: nuclear receptor coactivator 4
NEDD4L: NEDD4-like E3 ubiquitin ligase
NK cells: natural killer cells
NRF2: nuclear factor erythroid 2–related factor 2
OA: oleic acid
ORR: objective response rate
PARL: presenilin-associated rhomboid-like protein
PD-1: programmed death-1
PD-L1: programmed death-ligand 1
PE: phosphatidylethanolamine
PE-AA: phosphatidylethanolamine bearing an arachidonoyl moiety
PE-AdA: phosphatidylethanolamine bearing an adrenoyl moiety
PKCβII: protein kinase C βII
PL•: phospholipid radical
PLOH: phospholipid alcohol
PLOO•: phospholipid peroxyl radical
PLOOH: phospholipid hydroperoxide
PLGA: poly(lactic-co-glycolic acid)
PFS: progression-free survival
PUFA-PLs: polyunsaturated fatty acid–containing phospholipids
ROS: reactive oxygen species
RSL3: RAS selective lethal 3
RCC: renal cell carcinoma
SBRT: stereotactic body radiation therapy
SCD1: stearoyl-CoA desaturase 1
SMURF2: SMAD-specific E3 ubiquitin ligase 2
SOCS2: suppressor of cytokine signaling 2
SOD: superoxide dismutase
SQS: squalene synthase
STAT3: signal transducer and activator of transcription 3
STEAP3: six-transmembrane epithelial antigen of the prostate 3
TCA cycle: tricarboxylic acid cycle
Tf: transferrin
TfR1: transferrin receptor 1
TME: tumor microenvironment
Tregs: regulatory T cells
TKI: tyrosine kinase inhibitor
TNBC: triple-negative breast cancer
TRPML1: transient receptor potential mucolipin 1
TXNRD1: thioredoxin reductase 1
UPR: unfolded protein response
UPS: ubiquitin–proteasome system
VEGFR: vascular endothelial growth factor receptor
VK: vitamin K
VKH₂: vitamin K hydroquinone
